# A New Protein Superfamily: TPPP-Like Proteins

**DOI:** 10.1371/journal.pone.0049276

**Published:** 2012-11-14

**Authors:** Ferenc Orosz

**Affiliations:** Institute of Enzymology, Research Centre for Natural Sciences, Hungarian Academy of Sciences, Budapest, Hungary; University of South Florida College of Medicine, United States of America

## Abstract

The introduction of the term ‘Tubulin Polymerization Promoting Protein (TPPP)-like proteins’ is suggested. They constitute a eukaryotic protein superfamily, characterized by the presence of the p25alpha domain (Pfam05517, IPR008907), and named after the first identified member, TPPP/p25, exhibiting microtubule stabilizing function. TPPP-like proteins can be grouped on the basis of two characteristics: the length of their p25alpha domain, which can be long, short, truncated or partial, and the presence or absence of additional domain(s). TPPPs, in the strict sense, contain no other domains but one long or short p25alpha one (long- and short-type TPPPs, respectively). Proteins possessing truncated p25alpha domain are first described in this paper. They evolved from the long-type TPPPs and can be considered as arthropod-specific paralogs of long-type TPPPs. Phylogenetic analysis shows that the two groups (long-type and truncated TPPPs) split in the common ancestor of arthropods. Incomplete p25alpha domains can be found in multidomain TPPP-like proteins as well. The various subfamilies occur with a characteristic phyletic distribution: e. g., animal genomes/proteomes contain almost without exception long-type TPPPs; the multidomain apicortins occur almost exclusively in apicomplexan parasites. There are no data about the physiological function of these proteins except two human long-type TPPP paralogs which are involved in developmental processes of the brain and the musculoskeletal system, respectively. I predict that the superfamily members containing long or partial p25alpha domain are often intrinsically disordered proteins, while those with short or truncated domain(s) are structurally ordered. Interestingly, members of this superfamily connected or maybe connected to diseases are intrinsically disordered proteins.

## Introduction

The TPPPs, a new eukaryotic protein family, has recently been identified [1], [2]. Its first member, the Tubulin Polymerization Promoting Protein, TPPP/p25, was originally found as a brain-specific protein, p25alpha, with unknown function [3]. It is mainly expressed in differentiated oligodendrocytes [4]–[7]. This small, basic, unstructured protein promotes tubulin polymerization into normal and double-walled microtubules and induces their bundling [8]–[10]. It exhibits Microtubule Associated Protein (MAP)-like function by the stabilization of the microtubular network [10]–[12]. Under pathological conditions, TPPP/p25 is enriched in glial and neuronal inclusions in synucleinopathies as Parkinson's disease and multiple system atrophy [13], [14]. Recently, it has also been suggested that TPPP/p25 may work as a protective factor for cells against the damage effects of the accumulation of abnormal forms of prion protein [15].

There are three TPPP paralogs in the human genome; denoted as TPPP/p25, TPPP2/p18 and TPPP3/p20 (shortly TPPP1, TPPP2 and TPPP3, respectively), indicating their molecular mass [1]. TPPP3 but not TPPP2 shares the MAP-like features of TPPP1. The common C-terminal part of the three proteins (55–219 amino acids in TPPP1) is denoted as *p25alpha domain*, Pfam05517 or IPR008907, which corresponds practically to the whole sequence of TPPP2 or. There are no data about the function of these proteins except two human paralogs which are involved in developmental processes of the brain (TPPP1) [7], [12] and the musculoskeletal system (TPPP3) [16], respectively.

In this paper I have investigated the conservation of this protein/gene family and the occurrence of the p25alpha domain in a systematic bioinformatics study. I have denoted the proteins/genes containing the p25alpha domain as “*TPPP-like*” proteins/genes and characterized them from protists to vertebrates.

## Methods

### Database homology search

Accession Numbers of protein and EST sequences refer to the NCBI RefSeq and GenBank databases, respectively, except if otherwise stated.

The database search was started with an NCBI blast search using the sequences of human TPPP proteins (NP_008961; NP_776245; NP_057048). BLASTP or TBLASTN analysis [17] was performed on complete genome sequences and EST collections available at the NCBI website (http://www.ncbi.nlm.nih.gov/BLAST/). Even the hits when the BLAST E-score was higher than 1e^−10^ but less than 1 were investigated whether they can be considered as TPPP proteins. The reciprocal best-hit approach [18], [19] helped to reveal 1∶1 orthologies in some of these cases. Similar search was carried out on JGI databases (http://genome.jgi-psf.org/). Further sequences were identified at http://www.ncbi.nlm.nih.gov/Traces/home/, at the TBestDB page (http://tbestdb.bcm.umontreal.ca/) [20], at the GeneDB page (http://www.genedb.org/) [21] and at the page of the multicellularity project [22], http://www.broadinstitute.org/annotation/genome/multicellularity_project/MultiHome.html. Additionally, the sequences of several other TPPP orthologs were used for search. Generally, if a TPPP was found in a phylogenetic unit then the sequence of it was used as a query within the same unit. For example, the sequence of the *Chlamydomonas reinhardtii* FAP265 protein (XP_001695016) was used to find homologs among Archaeplastida. In the case of apicortins, the sequences of XP_002111209 (*Trichoplax adhaerens*) and XP_001609847 (*Babesia bovis*) were used as queries.

In the case of other multidomain proteins a higher threshold (1e^−2^) was used but the reciprocal best-hit approach cannot be applied. Moreover, the EBI InterPro (http://www.ebi.ac.uk/interpro/) [23], the Pfam protein families (http://pfam.sanger.ac.uk/) [24] and the CDD (http://www.ncbi.nlm.nih.gov/Structure/cdd/cdd.shtml) [25] databases were checked for proteins possessing p25alpha domain not detected by BLAST. Table 1 reports how many sequences total were found for the different subfamilies.

**Table 1:** Number of the identified TPPP-like proteins/ESTs.

Domain	Long p25alpha	Truncated p25alpha	Short p25alpha	Short p25alpha	Partial p25alpha	Partial p25alpha
Protein	Long-type TPPP	Truncated TPPP	Short-type TPPP	Multidomain proteins		Apicortin
**Total number**	**212 (55)**	**21**	**46 (5)**	**18**	**15 (5)**	**18 (1)**
***Opisthokonta***	**205 (48)**	**21**			**4**	**2**
Choanomonada	2				2	
Metazoa	200 (48)	21				1
Vertebrata	148 (47)					
Fungi	3				2	1
***Amoebozoa***					**1 (1)**	
***Apusozoa***					**1**	
***Archaeplastida***	**3 (3)**		**9 (2)**	**10**	**3 (1)**	**1 (1)**
Glaucophyta	1 (1)					
Chloroplastida	2 (2)		9 (2)	10	3 (1)	1 (1)
Chlorophyta			7	10	2	
Charophyta	2 (2)		2 (2)		1 (1)	1 (1)
***Chromalveolata***			**26**	**6**		**15**
Stramenopiles				6		
Alveolata			26			15
***Rhizaria***			**1 (1)**			
***Excavata***	**4 (4)**		**10 (2)**	**2**	**6 (3)**	
Fornicata					2	
Jakobida	3 (3)				2 (2)	
Malawimonas	1 (1)					
Preaxostyla					1 (1)	
Heterolobosea				2	1	
Euglenozoa			10 (2)			

The numbers of ESTs are in parenthesis.

Structural similarities were investigated by the PDBeFold (Structure Similarity) server (http://www.ebi.ac.uk/msd-srv/ssm/cgi-bin/ssmserver) [26].

### Alignments and phylogenic analysis

The phylogenetic classification and nomenclature applied in Adl et al. [27] is used through the paper. For higher level of classification, three megagroups and six supergroups are considered [28], [29]: unikonts (Opisthokonta+Amoebozoa); photosynthetic megagroup (Archaeplastida+Chromalveolata+Rhizaria); Excavata.

Multiple alignments of sequences were done by the ClustalW program [30]. Multiple sequence alignments used for constructing phylogenetic trees are shown in Figure S1 and 2. Bayesian analysis using MrBayes v3.1.2 [31] was performed to construct phylogenetic trees. Default priors were used. The Poisson model [32] was used assuming equal rates across sites. If gamma correction for different rates were incorporated no significantly different results were obtained. Two independent analyses were run with three heated and one cold chain (temperature parameter 0.2) for generations as indicated in the Figure legends, with a sampling frequency of 0.01 and indicated numbers of generations were discarded as burn-in. The two runs converged in all cases. The trees were drawn using the program Drawgram of the Phylip package version 3.68 [33].

### Prediction of unstructured regions

Sequences were submitted to the IUPRED server freely available at http://iupred.enzim.hu/ [34], [35]. POODLE-L, optimized for the identification of long disordered regions [36] was also used. This server is also freely available at http://mbs.cbrc.jp/poodle/poodle.html.

## Results and Discussion

### Grouping of TPPP-like proteins

**Figure 1:**
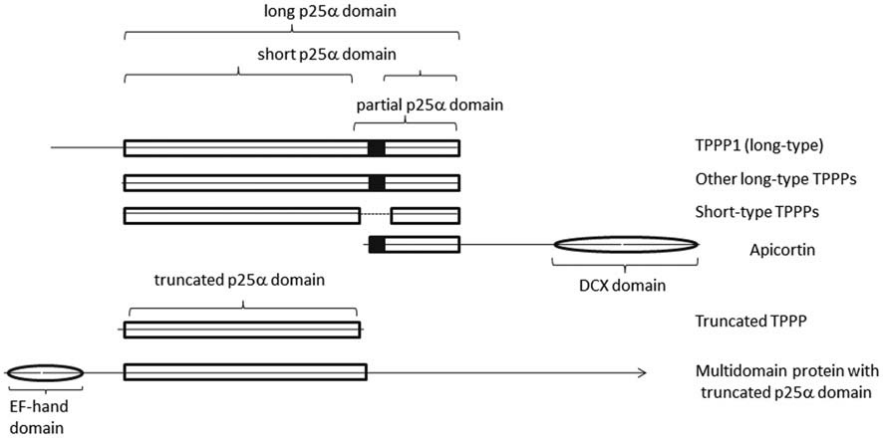
Graphical representation of the different types of architectures of TPPP-like proteins. The proteins are quasi-aligned, i.e., the length and the position of the domains correspond to the real situation. White boxes and ovals represent p25alpha domains and other kind of domains, respectively. Black squares show the position of the Rossmann-like motif. The dotted line in short-type TPPP represents the position of amino acids being present in long-type TPPPs but missing in short-type ones. Apicortin is the *T. adhaerens* one (XP_002111209); the multidomain proteins are represented by XP_003063447 of *M. pusilla*. The arrow at its end indicates that only the first half of the protein is shown on the figure. The length of the truncated domain is 100 amino acids in this protein.

**Figure 2:**
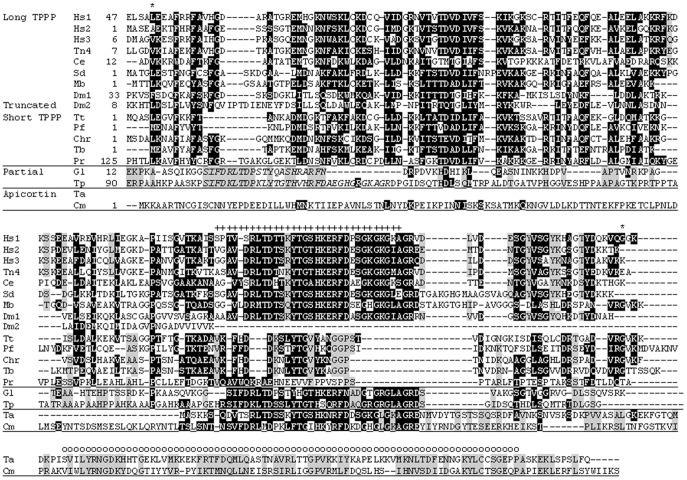
Multiple sequence alignment of several TPPP-like proteins by ClustalW. The alignment was refined manually. Long type TPPPs: *Hs1, Homo sapiens* TPPP1/p25 (NP_008961); *Hs2, Homo sapiens* TPPP2/p18 (NP_776245); *Hs3, Homo sapiens* TPPP3/p20 (NP_057048); *Tn4, Tetraodon nigroviridis* TPPP4 (CAF95233); *Dm1, Drosophila melanogaster* CG4893 (NP_648881); *Ce, Caenorhabditis elegans* C32E8.3 (NP_491219); *Sd, Suberites domuncula* (GH560390); *Mb, Monosiga brevicollis* (Monbr1/23057). (The *M. brevicollis* hypothetical protein was identified at http://genome.jgi-psf.org/Monbr1/Monbr1.home.html.) Truncated TPPP: *Dm2, Drosophila melanogaster* CG6709 (NP_648370). Short type TPPPs: *Tt, Tetrahymena thermophila* (XP_001023601); *Pf, Plasmodium falciparum* (XP_001350760); *Chr, Chlamydomonas reinhardtii* FAP265 (XP_001695016); *Tb, Trypanosoma brucei* (XP_844424); *Pr*, *Phytophthora ramorum* (phyra80518). Apicortins: *Ta, Trichoplax adhaerens* (XP_002111209); *Cm, Cryptosporidium muris* (XP_002139161). Proteins with several partial p25alpha domains: *Gl, Giardia lamblia* (XP_001705540); *Tp, Trimastix pyriformis* TPE00006173 (EC840067*). Amino acid residues identical and similar in one or more subfamilies are indicated by gray and black backgrounds, respectively. The asterisks indicate the beginning and the end of the p25alpha domain of the long and short TPPPs. The letters *x* and *o* label the partial p25alpha and the DCX domains, respectively. The additional partial p25alpha domains, present only in *G. lamblia* and *T. pyriformis*, are labeled by bold and italic letters.

TPPP-like proteins involve TPPPs and other proteins possessing one or more complete or partial p25alpha domain, Pfam05517 or IPR008907 (cf. Fig. 1 and 2). It is *not* a structural domain but was generated automatically from a sequence alignment from Prodom 2004.1 for the Pfam-B database (http://pfam.sanger.ac.uk/family/PF05517). The whole p25alpha domain of 140–160 amino acids can be found in TPPPs [1], [2], [9], [37]. TPPPs occur in two main different types, as *short-* and *long-type* ones [38]. Short- and long-type TPPPs, containing a *short* and *long* p25alpha domain, respectively, are different but paralogous proteins [39]. The C-terminal end of the short-type TPPPs is incomplete. Long-type TPPPs contains here a very conservative sequence of 31–32 amino acids. This part occurs independently from the whole domain as well, mostly in unicellular eukaryotes [38], and was denoted as *partial* p25alpha domain. The most characteristic part of this partial domain is the GXGXGXXGR Rossmann-like motif. In some cases the whole C-terminal part (i.e., the partial p25alpha domain) is missing. This kind of domains and proteins are first described in this paper and are named *truncated* p25alpha domain and TPPP, respectively. Additionally, there are multidomain proteins containing other domains than p25alpha as well.

### Long-type TPPPs

Long-type TPPPs possess the whole p25alpha domain. They eventuate in all the three phylogenetic megagroups (i.e. unikonts, the photosynthetic megagroup and Excavate) and are the most abundant in Opisthokonta, especially in animals (Metazoa) (cf. Table 1). Long-type TPPPs can be found in each animal genome sequenced except that of *T. adhaerens*. Vertebrates contain at least three long-type paralogs (TPPP1, TPPP2, TPPP3) due to the ancient two rounds genome duplication occurred in the vertebrate lineage.TPPP1 possesses an N-terminal tail of about 50 amino acids, not part of the p25alpha domain, which is missing in TPPP2 and TPPP3 (cf. Fig. 1 and 2). The fourth paralog (TPPP4) was either lost or retained only in fishes [39]. Other animals (Metazoa) and choanomonada, the unicellular sister group of Metazoa, contain generally only one copy of this protein, although species specific duplication happened in some cases. It occurs only in flagellated fungi (Chytridiomycota and Allomyces) and is absent in Amoebozoa.

It is rather rare in the photosynthetic megagroup (Archaeplastida+Rhizaria+Chromalveolata). It can be found at EST level in the Glaucophyta *Cyanophora paradoxa* and in two land plants *Hordeum vulgare* (barley) and *Oryza sativa* (rice). In Excavata, it is present in two phyla, Jakobida and Malawimonas. (The long-type TPPPs are listed in [38] and [40]).

### Truncated TPPPs

These proteins are identified in this paper. They are discussed after the long-type TPPPs since it seems that they evolved by the loss of the last exon of long-type TPPPs (see later). They occur only in some animals, mostly in Endopterygota, insects undergoing on metamorphosis, e.g., flies, butterflies, ants, beetles. In some cases it might happen that these proteins are artifacts due to incomplete sequencing but in the case of flies (Diptera), including all the twelve Drosophila species, where the whole genomes are known, it can be excluded. These proteins are listed in Table 2. In each case, the given species possesses a long-type TPPP as well.

**Table 2:** List of truncated TPPPs.

Phylogenetic group	Species	ID	GI	Source
**Arthropoda**				
Hexopoda Insecta Endopterygota				
	*Drosophila melanogaster*	NP_648370	24662040	RefSeq
	*Drosophila sechellia*	XP_002029959	195326485	RefSeq
	*Drosophila simulans*	XP_002084342	195589197	RefSeq
	*Drosophila erecta*	XP_001972246	194868209	RefSeq
	*Drosophila yakuba*	XP_002094265	195493080	RefSeq
	*Drosophila ananassae*	XP_001957775	194750915	RefSeq
	*Drosophila willistoni*	XP_002062203	195428283	RefSeq
	*Drosophila persimilis*	XP_002025402	195169178	RefSeq
	*Drosophila pseudoobscura*	XP_001353716	125979367	RefSeq
	*Drosophila mojavensis*	XP_002007566	195126208	RefSeq
	*Drosophila virilis*	XP_002047114	195376667	RefSeq
	*Drosophila grimshawi*	XP_001983728	195012698	RefSeq
	*Anopheles gambiae*	XP_556944	57918257	RefSeq
	*Culex quinquefasciatus*	XP_001862283	170052572	RefSeq
	*Camponotus floridanus*	EFN74475	307190439	GenBank
	*Solenopsis invicta*	EFZ11240^1^	322784183	GenBank
	*Danaus plexippus*	EHJ66593	357609707	GenBank
	*Tribolium castaneum*	EFA09619	270013171	GenBank
		EEZ98749	270002302	GenBank
Chelicerata Arachnida Acari				
	*Ixodes scapularis*	XP_002404704	241731346	RefSeq
	*Metaseiulus occidentalis*	XP_003742023	391335280	RefSeq
**Platyhelminthes**				
Trematoda				
	*Clonorchis sinensis*	GAA47940^1^	358339980	RefSeq

1 Phylogenetic analysis makes questionable whether EFZ11240 and GAA47940 belong to this group.

### Short-type TPPPs

Short-type TPPPs contain a short p25alpha domain, which corresponds to the whole or major part of their sequences (cf. Fig 1 and 2). They are absent in unikonts (Opisthokonta and Amoebozoa) but can be found in all other supergroups (cf. Table 1). In the Archaeplastida supergroup short-type TPPP seems to be common in Clorophyta (green algae), in various classes such as Chlorophyceae (*Chlamydomonas reinhardtii, Volvox carterii*), Prasinophyceae (*Micromonas pusilla, Ostreococcus* spp.) and Trebouxiophyceae (*Chlorella variabilis*). In Charophyta, which includes also land plants, only the species *Triticum aestivum* (wheat) and *O. sativa* (rice) contain short-type TPPP as EST. The latter one is especially important since *O. sativa* is the only species which is known to contain both long- and short-type TPPP genes.

This protein is also widely distributed in all the three phyla of Alveolata (Apicomplexa, Ciliophora, Dinozoa), representing its occurrence in the Chromalveolata supergroup (cf. Table S1). In Rhizaria only one example is known (*Paracercomonas marina*); however, for this supergroup generally much less sequence data is known than for other ones. Finally, in Excavata, short-type TPPP is common in the phylum of Euglenozoa including Kinetoplastea, Diplonemea and Euglenida.

Interestingly, in many species more paralogs of short-type TPPP can be found. This is the situation in Clorophyta, Alveolata and Euglenozoa as well. As the phylogenetic analysis has shown (see later), these multiple occurrences are the results of species and lineage specific duplications. (The short-type TPPPs are listed on Figure S4.)

### TPPP-like multidomain proteins containing short/truncated p25alpha domain(s)

In addition to the incidences of short p25alpha domain in short-type TPPPs, it occurs as a part of larger proteins. The length of the p25alpha domains in these proteins range between about 70 and 140 amino acids thus it is not unambiguous whether they can be considered as truncated or short domains. The first half of the p25alpha domain is always present but the length of the C-terminal part varies. This kind of occurrence happens mostly in two photosynthetic supergroups, Archeaplastida and Chromalveolate (cf. Table 1). They are represented by several green algae of the phylum of Clorophyta, and various members of the stramenopiles, respectively (Table 3).

**Table 3:** List of multidomain proteins/ESTs containing short/truncated p25alpha domain.

Phylogenetic group	Species	ID	Source	Number of short p25alpha domains	Other domain/motif	
					Name	CDD
**Archaeplastida**						
	*Chlamydomonas reinhardtii*	XP_001691800 (GI:159467228)	RefSeq	2	EFh	28933
	*Volvox carteri*	XP_002948912 (GI:302834700)	RefSeq	2	EFh	28933
	*Micromonas pusilla*	XP_003058058 (GI:303277529)	RefSeq	3	EFh COG4942	28933 34550
		XP_003063447 (GI:303288317)	RefSeq	1	EFh	28933
		XP_002506378 (GI:255088912)	RefSeq	2	EFh	208857
		XP_002507907 (GI:255081370)	RefSeq	2	EFh	28933
		XP_003061031 (GI:303283480)	RefSeq	2	-	-
	*Chlorella variabilis*	EFN57882 (GI:307109645)	GenBank	2	-	-
	*Coccomyxa subellipsoidea*	EIE25016 (GI:384251539)	GenBank	2	EFh	-
	*Ostreococcus lucimarinus*	XP_001421186 (GI:145353793)	RefSeq	2	-	-
**Chromalveolata**						
Stramenopiles	*Albugo laibachii*	CCA17632 (GI:325183175)	GenBank	1	P-loopNTPase DEXDc HELICc	208973 197756 28960
	*Ectocarpus siliculosus*	CBN75312 (GI:299117356)	GenBank	1	Znf BBOX IQ	206793 210118
		CBJ49059 (GI:298705751)	GenBank	1	zf-SNAP50_C Znf BBOX IQ WW	204865 206793 210118 206869
	*Phytophthora infestans*	XP_002905233 (GI:301112308)	RefSeq	1	Znf BBOX IQ COG5022	206793 210118 34627
		XP_002907084 (GI:301118713)	RefSeq	1	Mcp5_PH	206947
	*Phytophthora sojae*	EGZ26181 (GI:348686366)	GenBank	1	Znf BBOX IQ COG5022	206793 210118 34627
**Excavata**						
Heterolobosea	*Naegleria gruberi*	XP_002683090 (GI:291001047)	RefSeq	1	Kelch	207702
		XP_002682916	RefSeq	1	PLN02919 PTPc	29029 206804

These kinds of larger proteins of Clorophyta species contain the short p25alpha domain generally in duplicate but XP_003078535 of *Ostreococcus tauri* and XP_003063447 of *M. pusilla* contain only one copy. Some of them possess no other domain but their sequence are longer than usually (*M. pusilla* XP_003061031, *Ostreococcus lucimarinus* XP_001421186), while others possess additionally an *EF-hand* domain as well (*Ch. reinhardtii* XP_001691800, *V. carterii* XP_002948912, *M. pusilla* XP_002506378, XP_003063447 and XP_002507907). XP_003058058 of *M. pusilla* possesses the short p25alpha sequence in triplicate, an *EF-hand* region and *COG4942* domain. The function of EF-hands is generally the participation in Ca^2+-^binding, COG4942 is a membrane-bound metallopeptidase domain.

These kinds of proteins of flagellated stramenopiles, as *Ectocarpus siliculosus* and various *Phytophthora* species, contain always only one incomplete p25alpha domain, the length of which is less than the half of the whole sequence. In some cases a short sequence similar to a fragmentary “partial p25alpha domain” can also be found in these proteins, before (in *Phytophthora* species) or after (in *E. siliculosus*) the short p25alpha domain. A fragmentary protein in *Aureococcus anophagefferens* shows high similarity to the *E. siliculosus* one. In much longer proteins (900–1500 aa) other domains also occur, the most often *Znf BBOX* (B-Box-type zinc finger) and *IQ* ones. The IQ motif, an extremely basic unit of about 23 amino acids, serves as a Ca^2+-^independent binding site for different EF-hand proteins including the essential and regulatory myosin light chains, calmodulin, and calmodulin-like proteins. Znf BBOX is a zinc binding domain. Both domains occur in the following proteins which contain sometimes another domain as well: *Phytophthora infestans* XP_002905233 (and *COG5022* domain - myosin heavy chain); *E. siliculosus* CBN75312 and *E. siliculosus* CBJ49059 (and *WWP* or *Rsp5* domain). The *P. infestans* XP_002907084 possesses a pleckstrin homology and a *Mcp5_PH* domain beside the short p25alpha one. Another stramenopile protein, CCA17632 of *Albugo laibachii*, which is an RNA helicase, also contains a short p25alpha domain.

Finally, an Excavata species, the Heterolobosea *Naegleria gruberi* has two proteins of this kind of composition, XP_002683090 and XP_002682916, which contain one (Kelch) or two (PTPc and PLN02919) additional domains, respectively. These domains are generally related to various enzymatic functions as galactose oxidase (Kelch), ascorbate-dependent monooxygenase (PLN02919) and dual-specificity (Ser/Thr and Tyr) phosphatase (PTPc).

### Proteins with partial p25alpha domain(s)

The partial p25alpha domain, with or without the Rossmann-like motif, can be found in many organisms, in all megagroups, occurring independently from the other parts of the p25alpha domain (Table 4). They occur mostly but not exclusively in protists. In the majority of the cases, these proteins contain more than one copies of this partial p25alpha domain. Only one copy can be found in two choanoflagellate proteins, in *Monosiga brevicollis* (XP_001750206) and *Salpigoeca rosetta* (PTSG_03448). Both of them contain also the Rossmann-like motif. Fungal long-type TPPPs contain an additional partial p25alpha domain as well. An EST sequence from *Lolium perenne* (GR509039) indicates its presence in land plants. In the stramenopile *A. anophagefferens* the domain is coupled with a *WD40* repeat-like domain.

**Table 4:** List of proteins/ESTs but apicortins containing partial p25alpha domain.

Phylogenetic group	Species	ID	Source	Number of partial p25alpha domains	Rossmann-like motif
**Opisthokonta**					
Choanomonada	*Monosiga brevicollis*	XP_001750206 (GI:167537072)	RefSeq	1	yes
	*Salpigoeca rosetta*	EGD82798 (GI:326437228)	GenBank	1	yes
Fungi	*Batrachochytrium dendrobatidis*	EGF79566 (GI:328769522)	GenBank	2	yes, no
	*Spizellomyces punctatus*	SPPG_08463	Broad Institute^1^	2	yes
**Amoebozoa**					
Mycetozoa	*Hyperamoeba dachnaya*	EC854006* (GI: 110160603)	GenBank	3	yes
**Apusozoa**	*Thecamonas trahens*	AMSG_02233	Broad Institute^1^	4	yes
**Archaeplastida**					
Chloroplastida	*Chlamydomonas reinhardtii*	XP_001690551 (GI:159464643)	RefSeq	1	yes
	*Volvox carteri*	XP_002946586 (GI:302830039)	RefSeq	2	yes
	*Lolium perenne*	GR509039* (GI:300178892)	GenBank	1	yes
**Chromalveolata**					
Stramenopiles	*Aureococcus anophagefferens*	EGB10333 (GI:323454463)	GenBank	1	yes
	*Ectocarpus siliculosus*	CBN76131 (GI:299116327)	GenBank	1	no
	*Phytophthora infestans*	XP_002907772 (GI:301120089)	RefSeq	1	no
	*Phytophthora ramorum*	phyra80518 scaffold_50000026 draft genome v1.1	DOE JGI^2^	1	no
	*Phytophthora sojae*	EGZ29591 (GI: 348689777)	GenBank	1	no
**Excavata**					
Fornicata	*Giardia lamblia*	XP_001705540 (GI:159110572)	RefSeq	2	no, yes
		GL50581_3979	GiardiaDB^3^	2	no, yes
Jakobida	*Jakoba libera*	EC691986* (GI: 109799590)	GenBank	3	no
	*Seculamonas ecuadoriensis*	EC817264* (GI: 110123861)	GenBank	3	no
Preaxostyla	*Trimastix pyriformis*	EC840067* (GI:110146664	GenBank	3	yes
Heterolobosea	*Naegleria gruberi*	D2VER9_NAEGR (EFC44650)	UniProt	2	no

* Asterisks indicate ESTs.

1 http://www.broadinstitute.org;

2 http://genome.jgi-psf.org;

3 http://giardiadb.org/.

A special case of this independent occurrence is the apicortin where the partial p25alpha domain is combined with a DCX (Pfam03607, IPR003533) domain [38]. The DCX (doublecortin) domain is named after the brain-specific X-linked gene doublecortin [41]. Both domains (p25alpha ad DCX) are known to play an important role in the stabilization of microtubules ([8], [10] and [41], [42]) which suggests a similar function for apicortin. It occurs in two primitive opisthokonts, the placozoan *T. adhaerens* and the chytrid fungus, *Spizellomyces punctatus* (SPPG_06588) [20], [24]. An EST sequence from *Nicotiana tabacum* (AM844195) may indicate its presence in land plants. Recently available genomes and sequence data show that apicortin is a characteristic protein of the phylum of Apicomplexa. (The apicortins are listed in [43]).

**Figure 3:**
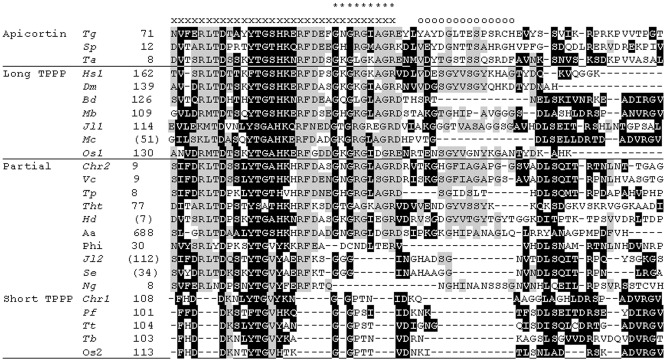
Multiple alignments of the C-termini of several short- and long-type TPPPs and partial p25alpha domains by ClustalW. The alignment was refined manually. Long type TPPPs: *Hs1, Homo sapiens* TPPP1/p25 (NP_008961); *Dm, Drosophila melanogaster* CG4893 (NP_648881); *Bd, Batrachochytrium dendrobatidis* (BDEG_06075); *Mb, Monosiga brevicollis* (Monbr1/23057); *Jl1, Jakoba libera* (EC692700*); *Mc, Malawimonas californiana* MCE00001955 (EC714749)*; *Os1, Oryza sativa* (CT849204*). Short type TPPPs: *Tt, Tetrahymena thermophila* (XP_001023601); *Pf, Plasmodium falciparum* (XP_001350760); *Chr1, Chlamydomonas reinhardtii* FAP265 (XP_001695016); *Tb, Trypanosoma brucei* (XP_844424); *Os2, Oryza sativa* (CT850609*). Apicortins: *Tg, Toxoplasma gondii* (EEA97769); *Sp, Spizellomyces punctatus* (SPPG_06588); *Ta, Trichoplax adhaerens* (XP_002111209). Proteins with partial p25alpha domain(s): *Chr2, Chlamydomonas reinhardtii* (XP_001690551); *Vc, Volvox carteri* (XP_002946586); *Tp, Trimastix pyriformis* TPE00006173 (EC840067*); *Tht, Thecamonas trahens* (AMSG_02233); *Hd, Hyperamoeba dachnaya* HDE00004089 (EC854006*); *Aa, Aureococcus anophagefferens* (EGB10333); *Phi, Phytophthora infestans* (XP_002907772); *Jl2, Jakoba libera* (EC691986*); *Se, Seculamonas ecuadoriensis* SEE00002453 (EC817264*); *Ng, Naegleria gruberi* D2VER9_NAEGR (EFC44650). Amino acid residues identical or similar in both short- *and* long-type TPPPs and in proteins containing partial p25alpha domain(s) are indicated by black background. Amino acid residues identical or similar in short- *or* long-type TPPPs and in proteins containing partial p25alpha domain(s) are indicated by grey background. The letter *x* labels the first 31–32 amino acids of partial p25alpha domains as in Fig. 2. Asterisks stands for the Rossmann-like motif (GXGXGXXGR). The letters *o* indicates an additional 14 aa sequence which is also missing in TPPP-like proteins which do not contain the Rossmann-like motif.

The green algae, *Ch. reinhardtii* and *V. carteri* share a significantly homologous protein, containing one and two partial p25alpha domains (including the Rossman-like motif), respectively, located at the N-terminal end of the *Chlamydomonas* (XP_001690551) and at both ends of the *Volvox* protein (XP_002946586). A similar domain arrangement can be found in some Excavata proteins, two of the *Giardia lamblia* and one of the *N. gruberi*. In the highly homologous *Giardia* proteins only the C-terminal domain contains the Rossmann-like motif (cf. Fig. 3), while this motif is lacking in the *Naegleria* (D2VER9_NAEGR) ones.

EST data revealed that the multiplication of the partial domain occurs in many other genomic sequences in various species: in the flagellated Amoebozoa, *Hyperamoeba dachnaya*; in the Excavata taxa, *Trimastix pyriformis*, *Seculamonas ecuadoriensis* and *Jakoba libera* (all in triplicate); and in the Apusozoa, *Thecamonas trahens* (alias *Amastigomonas*), in quadruplicate. The two Jakobida proteins (*Jakoba*, *Seculomonas*) miss the Rossmann-like motif.

The multiple alignment of the C-termini of short- and long-type TPPPs and the partial p25alpha domains (Fig. 3) suggests that the independent occurrence of this domain is not restricted to the 31–32 amino acid residues as suggested earlier [38], [43] and as indicated on Fig. 2. Instead, additional amino acids can be aligned with the C-termini of several short- and long-type TPPPs. However, this additional part was lost in animal and plant long-type TPPPs, as illustrated in the case of *H. sapiens*, *Drosophila melanogaster* and *O. sativa* TPPPs in Fig. 3. Other Opisthokonta TPPPs (in fungi and Choanomonada) and TPPPs in Excavata as well as short-type TPPPs preserved these amino acid residues. On the contrary, there is a 14 amino acid sequence in this “extended” partial p25alpha domain, following immediately the Rossmann-like motif, which is characteristic only for those TPPP-like proteins which contain this motif.

### Phylogenetic trees of TPPP-like proteins

**Figure 4:**
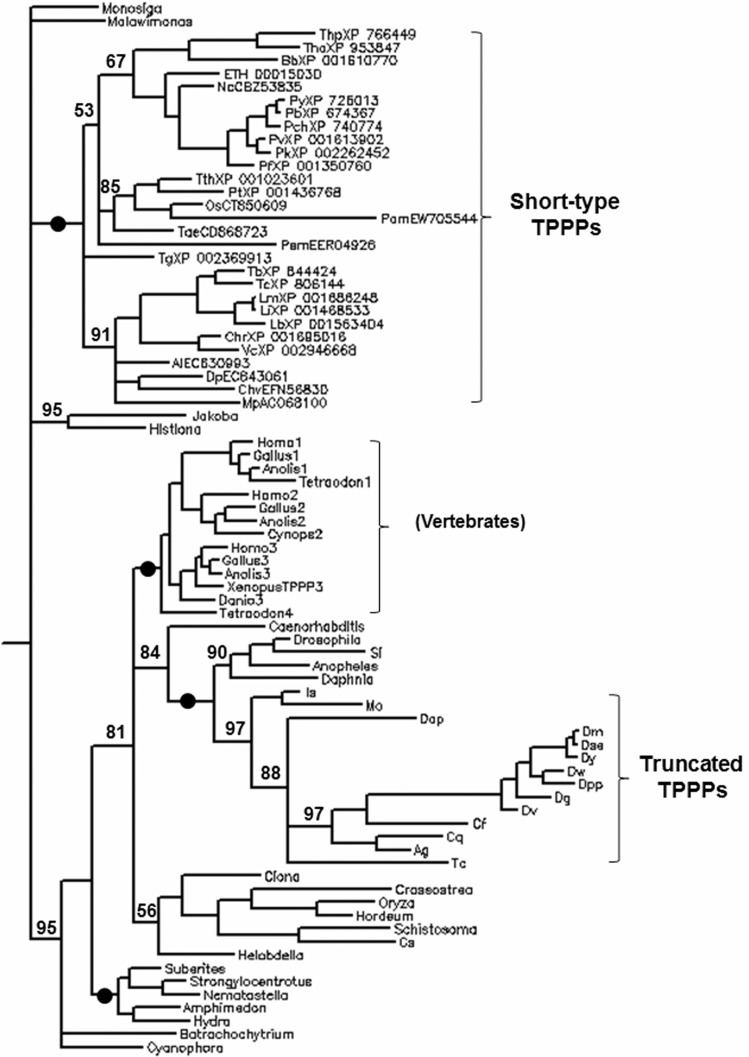
Phylogenetic tree of long-, short- and truncated TPPPs obtained by Bayesian analysis. Two independent analyses were run with three heated and one cold chain for 2×10^6^ generations, and 1.0×10^6^ generations discarded as burn-in. The numbers at the nodes represent clade credibility values; branches that received maximum support are indicated by full circles. For easier comparison, long-type TPPPs are labeled by name, truncated TPPPs by species code and short-type TPPPs by species code and accession number. All accession numbers are listed in Figure S1. Species codes are: ETH, *Eimeria tenella*; Os, *Oryza sativa*; Tae, *Triticum aestivum*; Thp, *Theileria parva*; Tha, *Theileria annulata*; Bb, *Babesia bovis*; Nc, *Neospora caninum*; Py, *Plasmodium yoelii*; Pb, *Plasmodium berghei*; Pch, *Plasmodium chabaudi*; Pv, *Plasmodium vivax*; Pk, *Plasmodium knowlesi*; Pf, *Plasmodium falciparum*; Tg, *Toxoplasma gondii*; Tb, *Trypanosoma brucei*; Tc, *Trypanosoma cruzi*; Lm, *Leishmania major*; Li, *Leishmania infantum*; Lb, *Leishmania brasiliensis*; Chr, *Chlamydomonas reinhardtii*; Vc, *Volvox carteri*; Al, *Astasia longa*; Dp, *Diplonema papillatum*; Chv, *Chlorella variabilis*; Mp, *Micromonas pusilla*; Pem, *Perkinsus marinus*; Tth, *Tetrahymena thermophila*; Pt, *Paramecium tetraurelia*; Pam, *Paracercomonas marina*; Cs, *Clonorchis sinensis*; Is, *Ixodes scapularis*; Mo, *Metaseiulus occidentalis*; Dap, *Danaus plexippus*; Dm, *Drosophila melanogaster*; Dse, *D. sechellia*; Dy, *D. yakuba*; Dw, *D. willistoni*; Dpp, *D. pseudoobscura*; Dv, *D. virilis* ; Dg, *D. grimshawi*; Cq, *Culex quinquefasciatus*; Ag, *Anopheles gambiae*; Tc, *Tribolium castaneum*; Si, *Solenopsis invicta*; Cf, *Camponotus floridanus*.

Fig. 4 shows a phylogenetic tree which contains the representatives of long-, short- and truncated TPPP. Of course, other TPPP-like proteins, which contain more than one domain, cannot be included in this analysis. Short- and long-type TPPPs are unambiguously separated, in accordance with the previous phylogenetic analysis [38]. It was concluded that short- and long-type TPPPs can be considered as different proteins which are in close relation (paralogs rather than orthologs). Interestingly, there is only one species where both kinds of TPPP genes can be found, *O. sativa*, whose translations correspond to hypothetical proteins of 156 and 185 amino acids, respectively. They show only 18% identity and 37% similarity in their sequence. In comparison, the short-type *O. sativa* (rice) protein share 55% of amino acids with that of the *T. aestivum* (wheat), while the long-type one is identical in 61% with that of the *H. vulgare* (barley) (Figure S3). It indicates that the presence of two kinds of TPPPs in *O. sativa* is not the result of an in-species gene duplication but the consequence of an event occurring in an early common ancestor of these corns, maybe in the common ancestor of eukaryotes. In this case we can consider short- and long-type TPPPs as “outparalogs” (for definition see Sonnhammer and Koonin [44]).

The detailed phyletic analysis of long-type TPPPs of Opisthokonts was carried out by Stifanic et al. [40] They concluded that although it was possible to reconstruct widely accepted phylogenetic trees, there were clear exceptions due to possible adaptation to environmental conditions, in the case of animals with cilia exposed to the aquatic environment. They did not discuss the case of the several other long-type TPPPs. As I showed earlier, they largely followed species phylogeny, at least with regard to the higher level taxonomic groups, with the exception of the place of the long-type TPPPs of land plants (*O. sativa* and *H. vulgare*) inside the bilaterian (the major animal) clade [38]. Due to the small number of long-type TPPPs in the photosynthetic megagroup, this fact is hard to be interpreted. Finally, the vertebrate TPPP paralogs (TPPP1, TPPP2, TPPP3) are grouped into different sub-clades within the long-type TPPP clade (Fig. 4).

Truncated TPPPs are embedded as a sub-clade into long-type TPPPs (Fig. 4). These arthropod proteins are more similar each other than to the corresponding long-type TPPPs in the same species. Their position on the tree supports that they evolved from the long-type TPPPs and can be considered as arthropod-specific paralogs of long-type TPPPs. The tree shows with very high clade credibility that the two groups (long-type and truncated TPPPs) split in the common ancestor of arthropods. The position of the only non-arthropod putative truncated protein from the flatworm, *Clonorchis sinensis*, suggests that it may not belong to this sub-family.

Phylogenetic tree of short-type TPPPs (Figure S4) mostly corresponds to the species phylogeny. A notable exception is that the relation of Euglenozoa and green algae (Clorophyta) is not well resolved which may be indicative of lateral transfer of the short-type TPPP gene between them. Considering the fact that Euglenozoa are the only Excavata group possessing short-type TPPP which is widely distributed in the photosynthetic megagroup, the donor was, if indeed lateral gene transfer occurred, likely from a branch of the algal lineage. In species where more paralogs of short-type TPPP can be found, as the phylogenetic analysis has shown, these multiple occurrences are the results of species (*Paramecium tetraurelia, Tetrahymena thermophila, Perkinsus marinus*) and lineage (*Leishmania*, Ciliophora, Apicomplexa) specific duplications.

Of course, multidomain proteins cannot be analyzed in this way, thus in this case only the short p25alpha domains were used in the analysis (Figure S5). Short-type TPPPs, whose whole sequence corresponds to this domain, were also involved in the building of the tree. Although most of the branches received poor supports (but the posterior probabilities were always higher than 50%], several conclusions can be done. Short-type TPPPs are well separated from the domains of the multidomain proteins. Algal and stramenopile domains form generally separated clades. The multiplied domains of various algal proteins are grouped by species showing the independent (in-species or in-protein) multiplications of these short p25alpha domains.

The phylogenetic tree built using the sequences of the partial p25alpha domains shows that short- and long-type TPPPs are separated, as in the case of the whole proteins (Figure S6). It refers to the short- and long-type *O. sativa* proteins as well, which is the only example for their common occurrence in the same species. The long-type TPPPs and apicortins, both groups containing the Rossmann-like motif, are also separated. These facts support the suggestion for the early separation of these proteins, probably in the last common ancestor of eukaryotes [38]. The multiplied domains of various protist proteins are grouped by species showing the independent (in-gene) multiplications of these partial p25alpha domains. In general, the Excavata and the unikont species containing these multiplied domains form independent clades.

### Summation of the phyletic distribution of TPPP-like proteins

As suggested recently, eukaryotes can be divided into three monophyletic megagroups: unikonts, Archaeplastida+Rhizaria+Chromalveolata, Excavata [28], [29]. The phyletic distribution of the long- and short-type TPPPs and that of the partial p25alpha domain containing proteins differs from each other (Table 1and Table S1). The most important difference is that the short-type TPPP (and the short type p25alpha domain) is not present in unikonts, i.e., in Opisthokonta and Amoebozoa. It is also missing in *T. trahens*, an Apusomonadida suggested recently as a sister group to Opisthokonta [45].

Opisthokonta is specific almost exclusively for the long-type TPPPs. Long-type TPPP is present in all the metazoan genomes known but *T. adhaerens* which contains instead a partial p25alpha domain as a part of apicortin. TPPP is absent in fungi but the flagellated ones, Chytridiomycota and Blastocladiomycota, which contain long-type TPPP orthologs, similarly to some choanoflagellates. There are a few proteins with partial p25alpha domain, including apicortins in *T. adhaerens* and in the fungus *S. punctatus*. In Amoebozoan genomes available neither short-type nor long-type TPPP was found, only the partial p25alpha domain was found in some flagellated *Hyperamoeba* species. The absence of TPPPs in Amoebozoa may be connected to the loss of flagellum in the majority of these taxa (e.g. *Dictyostelium discoideum, Entamoeba hystolica*). Truncated TPPPs, identified recently, can exclusively be found in some animals.

The “photosynthetic” megagroup (Archaeplastida+Rhizaria+Chromalveolata) is represented mainly by the short-type TPPP which is present in all three supergroups. In the case of Chromalveolata it holds for the monophyletic clade (stramenopiles and Alveolata including Apicomplexa, Ciliophora, and Dinozoa) but not for the HC group (Haptophyta and Cryptomonads), in which no TPPP-like protein was found, at least in the databases available. The apicomplexan species contain, beside the short form, also a partial p25alpha domain as part of apicortin. For Rhizaria only very few data are available but the biflagellated Rhizarian, *P. marina*, contains a short-type ortholog, while the amoeboid *Bigelowiella natans* seems to miss it, which supports the proposed connection between cilia/flagella and TPPP proteins [46].

The Archeaplastida (beside Excavata) shows the most multifarious picture concerning the distribution of these protein family members. Green algae contain short-type TPPPs, partial p25alpha domain containing proteins and multidomain proteins with more than one short p25alpha domains. Multidomain proteins can be found also in stramenopiles. A Glaucophyta (*C. paradoxa*) and several Charophyta (*Hordeum, Oryza*) contain long-type TPPP, at least at EST level. Moreover, beside the long-type TPPP, *O. sativa* contains also a short-type one. There are two examples for the occurrence of partial p25alpha domain as ESTs (*Lolium, Nicotiana*). It is quite interesting since land plants (e.g. *Arabidopsis*), which are fully sequenced, are known not to contain the members of this protein family.

In Excavata, according to the EST data available, both short- and long-type TPPPs and the partial domain are widely distributed. Euglenozoa, on one hand, Jakobida and Malawimonadidae, on the other hand, are characterized by the occurrence of the short and long form, respectively. Several proteins/genes in *Giardia*, *Trimastix*, *Naegleria* and the jakobida *Seculamonas* contain only the partial p25alpha domain but in duplicate or in triplicate (cf. Figure S7). *J. libera* also contains, beside the long-type TPPP, this form. The whole sequences of the ESTs containing the partial p25alpha domain in triplicate are rather similar, especially those of the two jakobids, and they are reciprocal best hits of each other's.

### Structural considerations

NMR structures are available only for a few long-type TPPPs: CE32E8.3 of *Caenorhabditis elegans* [47], TPPP2 of mouse [48] and of human [49], and human TPPP1 [50]. Comparing these structures with other PDB structures, weak similarity was found only with calmodulin and other calcium binding proteins, complexed not only with Ca but other bivalent cations (Mg, Mn, Zn) as well. It is not surprising since some, also very weak, sequence similarity exists among TPPPs and these proteins. Moreover, human TPPP1 was shown to be a Zn-binding protein [51].

The long N-terminal tail, present only in TPPP1, is fully disordered (∼50 aa). The further part of the molecules, present in all long-type TPPPs, is composed of two distinct regions. The C-terminal, sequentially conserved, part is unstructured (about ∼60 aa) in all cases. The middle, less conserved, region is more ordered. In the case of TPPP1 it is rather flexible; the other three proteins possess 5 α-helices in this part; human TPPP2 has also 2 β-sheets. This region corresponds to the first two coding exons, while the C-terminus to the third one, not only in human but in most of the long-type TPPPs [40]. The positions of the helices are conserved despite of the amino acid substitutions of this region. Interestingly, in the long-type TPPPs of the various Drosophila species, the first and the second exons are merged, i.e., an intron was lost.

**Figure 5:**
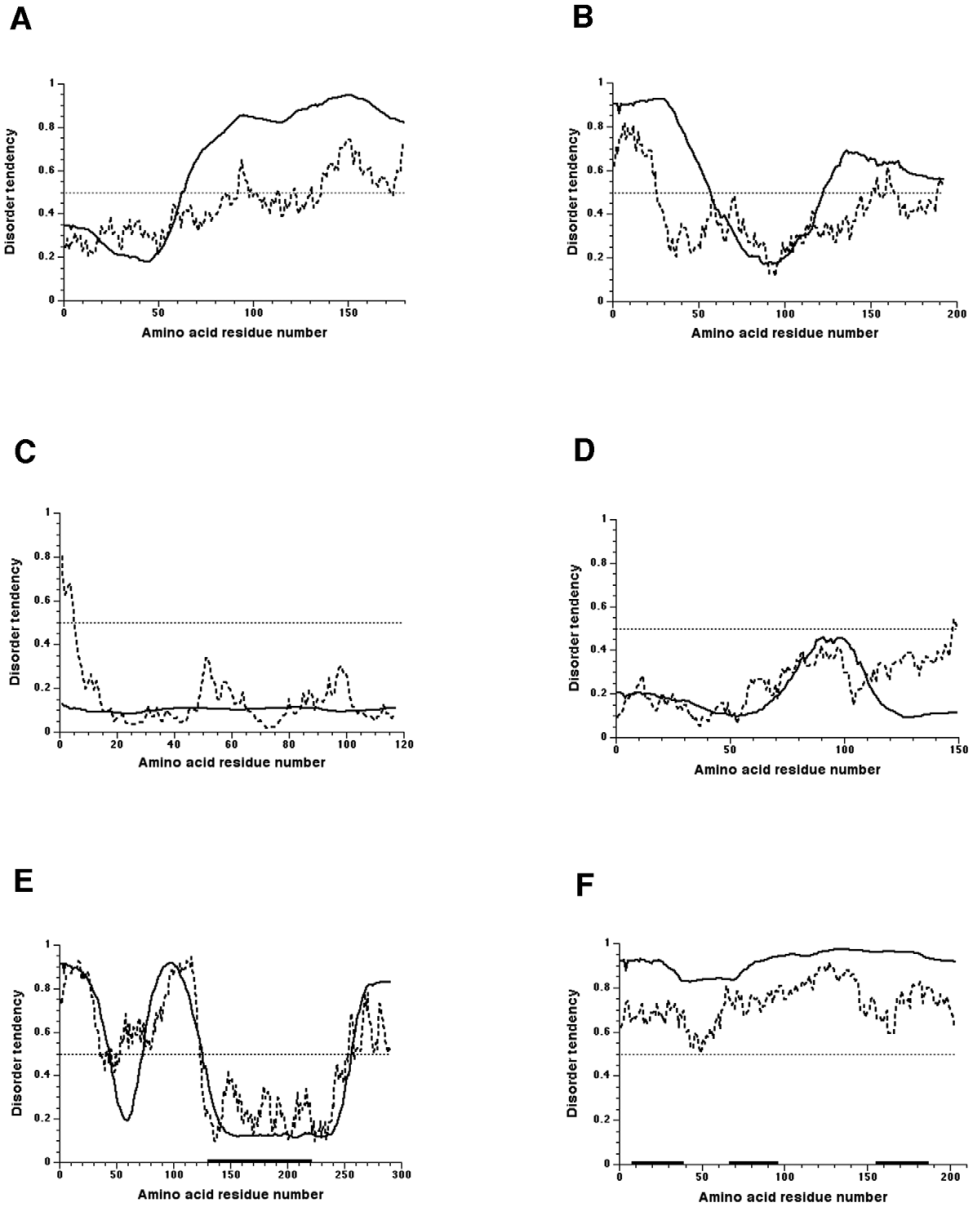
Disorder prediction of TPPP-like proteins using POODLE-L (solid line) and IUPRED (dotted line) predictors. Disorder prediction values for the given residues are plotted against the amino acid residue number. The significance threshold, above which a residue is considered to be disordered, set to 0.5, is shown. A) *C. elegans* (NP_491219), B) *D. melanogaster* CG4893 (NP_648881), C) *D. melanogaster* CG6709 (NP_648370), D) *T. thermophila* (XP_001023601), E) *Ph. ramorum* phyra80518, F) C) *H. dachnaya* HDE00004089 (EC854006*). The short (E) and partial (F) p25alpha domains are indicated by bold lines at the bottom of the plots.

The disordered regions of human TPPP1 have probably functional role since they were suggested to be responsible for the binding of the protein to microtubules [10], [49]. Since the structures of other family members are not available thus I used two protein disorder prediction methods (for recent reviews see [52], [53]) for getting a general overview of the order/disorder status of TPPP-like proteins. Examples are shown on Fig. 5 and Figure S7. On the basis of the predictions, the following conclusions can be drawn:

Long-type TPPPs have generally been predicted to be similar as established experimentally for the above mentioned cases. The C-termini of the TPPPs are predicted to be disordered, as well as the N-terminal tail of the *D. melanogaster* one. (Insect long-type TPPPs, including CG4893 of *D. melanogaster*, have an N-terminal tail, similarly to the N-terminus of human TPPP1.)

Short-type and truncated TPPPs are generally predicted to be ordered in their full length. The examples of *T. thermophila* and *Plasmodium falciparum* short-type as well as that of the *D. melanogaster* truncated proteins are shown. Their sequences correspond mostly to the ordered part of the long-type TPPPs. Importantly, if proteins contain more than one short/truncated p25alpha domains, all of these domains are predicted to be ordered (cf. Figure S7D).

Members of another class of TPPP-like proteins contain only (a) partial p25alpha domain(s), the sequence of which is very conservative and corresponds to the C-terminal part of long TPPPs. Characteristically, the partial p25alpha domain occurs in disordered proteins. Proteins containing this sequence in more than one copy are generally fully disordered (Fig. 5F and Figure S7E–G). In the special case of apicomplexan apicortins, it has recently been shown that they possess a disordered N-terminal tail and a shorter disordered linker between the partial p25alpha and DCX domains [42]. The microtubule binding function of these proteins was also suggested [38].

In conclusion, one can hypothesize that long-type TPPPs and proteins with partial p25alpha domain have a role in microtubule organization due their disordered character, while short-type and truncated TPPPs and proteins with short p25alpha domain may miss this function. Naturally, experimental verification of this hypothesis is needed.

Interestingly, members of this superfamily connected or maybe connected to diseases are intrinsically disordered proteins. Apicortins occur almost exclusively in apicomplexan parasites responsible for illnesses as malaria and toxoplasmosis. It was suggested that they are involved in the so called apical complex of these protists, which has important role in the pathogen-host interactions. A long-type TPPP (human TPPP1) was shown to be enriched in glial and neuronal inclusions in synucleinopathies as Parkinson's disease and multiple system atrophy [13], [14] and suggested to work as a protective factor for cells against the damage effects of the accumulation of abnormal forms of prion protein [15].

### Evolution of TPPP-like proteins

The TPPP gene was considered to be conserved in the genomes of ciliated/flagellated eukaryotes but to be absent from those that are non-ciliated [46]. (Eukaryotic cilia/flagella are organelles with a microtubule-based cytoskeleton called the axoneme.) Although the strength of this relationship seems to be slightly weakened since TPPP genes (but not yet proteins) were identified in a few land plants without these organelles [38] but the ancient origin of this protein family is supported by the ancient origin of the eukaryote cilia/flagella and by the fact that its members are widely distributed in the phylogenomic “supergroups” (Table 1). TPPP-like proteins can be found in taxa of all the six eukaryotic supergroups. As suggested recently, eukaryotes can be divided into three monophyletic megagroups: unikonts, photosynthetic megagroup, Excavata [28], [29]. The presence of a protein family in all megagroups is indicative of its very ancient origin except in the case of lateral transfer [54], [55]. Although in some cases lateral gene transfer might happen (see above), considering the wide phyletic distribution of TPPPs, I can suggest that long- and short-type TPPPs and the partial p25-alpha domain were present in the last common ancestor of eukaryotes. If we consider the present view of the eukaryote tree of life [28], [29], [56], we can conclude that the loss of short-type TPPP could occur in the common ancestor of the ‘unikonts’, which was followed by the loss of long-type TPPP in the common ancestor of Amoebozoa. On the other hand, the common ancestor of the ‘photosynthetic megagroup’ still contained all the three kinds of genes but the long-type TPPP could be lost in the ancestor of the SAR (stramenopiles, Alveolata, Rhizaria) group and preserved in Archaeplastida. Thus short- and long-type TPPPs are different proteins which are in close relation and can be can considered as “outparalogs”.

The truncated TPPPs evolved by the loss of the last exon of long-type TPPPs in some arthropods (Arthropoda), especially in Entopterygota (insects undergoing on metamorphosis). It occurs also in other Arthropoda subphylum, Chelicerata, in ticks and mites; and perhaps in a flatworm, *C. sinensis* but phylogenetic analysis does not support it. In the case of insect truncated TPPPs their common origin can be suggested since they are more similar to each other than to long-type TPPPs occurring in the same species. Interestingly, in Drosophila species, in contrast to their long-type TPPPs, where the N-terminal part of the proteins are coded by a single exon, and the C-terminal part by another one, truncated TPPPs preserved the intron separating their coding exons, similarly to the majority of long-type TPPPs.

The combination of short and partial p25alpha domains with various other domains has of special interest. Apicortin is a chimeric protein of partial p25alpha and DCX domains. Its evolution is enigmatic because of its very limited and specific phyletic occurrence: it is present only in few species except the phylum Apicomplexa. On the other hand, the DCX domain, which is common in Metazoa, was not found in the photosynthetic megagroup [57] except apicortins. These problems have been discussed in details recently [43]. The presence of this protein in two different phylogenetic megagroups (unikonts and the photosynthetic megagroup) is indicative of its ancient origin (the last common ancestor of eukaryotes) with general gene loss, except if lateral gene transfer occurred. The recent findings make more probable the first scenario [43].

The other multidomain proteins being present mostly on algae and stramenopiles seem to be of lineage specific origin.

## Supporting Information

Figure S1 Multiple sequence alignments of TPPP proteins by ClustalW used for constructing the phylogenetic tree on Fig. 4.
(DOC)

Figure S2 Multiple sequence alignments of TPPP-like proteins by ClustalW used for constructing the phylogenetic trees.
(DOC)

Figure S3 Multiple sequence alignment of *Triticum*, *Hordea* and *Oryza* TPPPs by ClustalW. The alignment was refined manually. Amino acid residues identical and similar in both long- and short-type TPPPs are indicated by black background. Amino acid residues identical and similar only in long- or short-type TPPPs are indicated by pink and blue backgrounds, respectively. The three pairs of amino acid residues identical and similar only in long- and short-type *Oryza* TPPPs are indicated by grey background.
(DOC)

Figure S4 Phylogenetic tree of the short-type TPPPs obtained by Bayesian analysis. Two independent analyses were run with three heated and one cold chain for 2×10^6^ generations, and 1.0×10^6^ generations discarded as burn-in. The numbers at the nodes represent clade credibility values; branches that received maximum support are indicated by full circles. Proteins and ESTs (labeled by asterisk) are indicated by species code and database accession number. ETH (*Eimeria tenella*) sequences were identified at http://www.genedb.org/. Species codes are: Os, *Oryza sativa*; Tae, *Triticum aestivum*; Thp, *Theileria parva*; Tha, *Theileria annulata*; Bb, *Babesia bovis*; Nc, *Neospora caninum*; Py, *Plasmodium yoelii*; Pb, *Plasmodium berghei*; Pch, *Plasmodium chabaudi*; Pv, *Plasmodium vivax*; Pk, *Plasmodium knowlesi*; Pf, *Plasmodium falciparum*; Tg, *Toxoplasma gondii*; Tb, *Trypanosoma brucei*; Tc, *Trypanosoma cruzi*; Lm, *Leishmania major*; Li, *Leishmania infantum*; Lb, *Leishmania brasiliensis*; Chr, *Chlamydomonas reinhardtii*; Vc, *Volvox carteri*; Al, *Astasia longa*; Dp, *Diplonema papillatum*; Chv, *Chlorella variabilis*; Mp, *Micromonas pusilla*; Pem, *Perkinsus marinus*; Tth, *Tetrahymena thermophila*; Pt, *Paramecium tetraurelia*; Pam, *Paracercomonas marina*.
(TIF)

Figure S5 Phylogenetic tree of the short p25alpha domains obtained by Bayesian analysis. Two independent analyses were run with three heated and one cold chain for 2.6×10^6^ generations and 2.1×10^5^ generations discarded as burn-in. Species codes are the same as in Fig. 4 and Figure S4. Further codes are: Ot, *Ostreococcus tauri*; Ol, *Ostreococcus lucimarinus*; Es, *Ectocarpus siliculosus*; Albugo, *Albugo laibachii*; Pr, *Phytophthora ramorum*; Pi, *Phytophthora infestans*; Ps, *Phytophthora sojae*; Ng, *Naegleria gruberi*. The Accession Numbers of proteins and ESTs (*) are listed in Figure S1. MD stands for “domains of multidomain proteins”.
(TIF)

Figure S6 Phylogenetic tree of the partial p25alpha domains obtained by Bayesian analysis. Two independent analyses were run with three heated and one cold chain for 1.1×10^6^ generations and 5.5×10^5^ generations were discarded as burn-in. Cr hominis and Cr parvum stand for *Cryptosporidium hominis* and *Cryptosporidium parvum, respectively;* Plasmodium for *Plasmodium falciparum*, and Tetrahymena for *Tetrahymena thermophila*. The Accession Numbers of proteins and ESTs (*) are listed in Figure S1.
(TIF)

Figure S7 Disorder prediction of TPPP-like proteins using POODLE-L (solid line) and IUPRED (dotted line) predictors. Disorder prediction values for the given residues are plotted against the amino acid residue number. The significance threshold, above which a residue is considered to be disordered, set to 0.5, is shown. A) *M. brevicollis* (Monbr1/23057); B) *S. domuncula* (GH560390); C) *P. falciparum* short-type TPPP (XP_001350760); D) *M. pusilla* EEH58009 (XP_003058058); E) *G. lamblia* (XP_001705540); F) *T. trahens* AMSG_02233; G) *T. pyriformis* TPE00006173 (EC840067*). The short (D) and partial (E–G) p25alpha and other (COG4942 and EF-hand) (D) domains are indicated by solid and dotted lines, respectively, at the bottom of the plots.
(TIF)

Table S1 Phyletic distribution of the TPPP-like proteins.
(DOC)
