# Supplementary material for: A New Protein Superfamily: TPPP-Like Proteins
Source: PLoS One. 2012 Nov 14;7(11):e49276. doi: 10.1371/journal.pone.0049276 (PMC3498115; doi:10.1371/journal.pone.0049276)
Supplement: Figure S1 — Multiple sequence alignments of TPPP proteins by ClustalW used for constructing the phylogenetic tree on Fig. 4. (DOC) [file pone.0049276.s001.doc]

CLUSTAL 2.1 multiple sequence alignment: long-, truncated- and short TPPPs (Fig. 4)

Monosiga Monbr1/23057 --------------MTTLKQVFEQYASFGAGSAATG------TPELDSAKFTKLCKETKLIS--KSLTTTDADLIFT--RVKAK-----------GQRKIGFAEFRS-ALEEVA-KK---------TGQDVSAVEAKVTRAGGPQSSG-TQADSGGVL-DRMTDTSQYTGSHKERFDSE-GHGKGLAGRDSTAKGTGHIPAVGGG----S-DLASHLDRSPA--NVRGVKK------

Jakoba EC692700 -------------MEDHLYDVFAQFCGFGDRSAVS---------EMDGARFAKLCKDTKLVC--SRFTRTDVDLVFA--KVKPK-----------TGRRINYEEFKD-ALALAGAQIWRNS----PPEEAFLKATDRVVENGNPKLSNTTASSTDEVL-EKMTDVNLYSGAHKQRFNED-GTGRGREGRDVIAKGGGTVASAGGSG-AVHDLSEI-TRSHL---NTGPSALSERVSP

Histiona EC851037 ---MS-----RQQHHKPLHDVFRSFAAFGAGSSAS----QNADVLLDGAKFVKLCKDCQLIG--SSFTVTDADLIFS--KVKPR-----------TERKIDFKQFRN-ALTLIA-EKMHPDM--DAETEGRRAVEDRILAHHGPQLSGVTAVEGDQAL-DRLTNEALFTGMYXARFEE-----------------------------------------------------------

Malawimonas EC714749 ??????????????????????????????????????????????????????????????????????????????????????????????KTARKINFAQFDK-ACHLLADAKG----------ISYDEFVAKALEAGGPAKVGVTQVAEDGIL-SKLTDASQYTGAHKERFDAD-GKGRGLAGRDHPVTG---------------DLSELLDRTDA---DVRGVKKA-----

Homo1 NP_008961 AGEGAAAS----PELSALEEAFRRFAVHGD--ARAT------GREMHGKNWSKLCKDCQVIDG-RNVTVTDVDIVFS--KIKGK-----------SCRTITFEQFQE-ALEELAKKRFKDKSS----EEAVREVHRLIE-GKAPIISGVTKAISSPTV-SRLTDTTKFTGSHKERFDPS-GKGKGKAGRVD--------LVDE------SGYVSGYKHAGTY--DQKVQGGK-----

Gallus1 XP_001231864 SHEGAMAG-----EISALEEAFRKFAIHGD--TRAT------GKEMHGKNWSKLCKDCQVIDG-KNVTITDVDIVFS--KIKGK-----------SSRTITFEQFKE-ALQELSKKRFKEKSD----EEAIQEIYKLIE-GKAPIISGVTKAISSPTV-SRLTDTSKFTGSHKERFDPS-GKGKGRAGRED--------LVDA------SGYVSGYKHAGTY--DHKVQGSK-----

Anolis1 XP_003222359 SHEG-VSGA----ELSALEEAFRKFAIHGD--TRAT------GKEMHGKNWSKLCKDCHVIDG-KNVTLTDVDIVFS--KIKGK-----------S-RTITYDQFKE-ALQELSKKRFKDKSN----EEAVQEMFKLIE-GKGPVISGVTKAISSPTV-SRLTDTTRFTGSHKERFDPS-GRGKGKAGRED--------LVDT------SGYVSGYKHAGTY--DHKVQGSK-----

Tetraodon1 CAG11971 TSEGGMGSSTP-VELTALEEAFRRFAIHGD--TRAT------GKEMHGKNWSKLCKDCGVIDG-KSITLTDVDIVFS--KVKKK-----------SCRNITYDEFKT-ALAELARKKYKEKSG----EEAEAEIFKLVE-GKSPIISGVTRAVASPTV-SRLTDTTKFTGSHKERFDST-GRGKGKAGRED--------IVDT------SGYVSGYKHRGTY--EKKV---------

Homo2 NP_057048 ---MASEA----------EKTFHRFAAFGE--SSSS------GTEMNNKNFSKLCKDCGIMDG-KTVTSTDVDIVFS--KVKAK-----------NARTITFQQFKE-AVKELGQKRFKGKSP----DEVLENIYGLME-GKDPATTGATKATTVGAV-DRLTDTSKYTGTHKERFDES-GKGKGIAGREE--------MTDN------TGYVSGYKGSGTY--DKKTK--------

Gallus2 XP_424853 ---MSG-----------LEESFRKFAVYGD--TAAS------GNNMTGKNFSKMCKECGVMDG-KAVTSTDIDIVFN--KVKTK-----------GARTINFVEFQQ-AMKEICVKRFKGKSP----EEALQAVYGLIE-GKEPSNVGTTKVAKVAGV-DRLTDTSKYTGSHKERFDES-GKGKGLAGRED--------LTDN------SGYVGAYKGAGTY--DKTH---------

Anolis2 XP_003224558 ---MAN-----------LESTFRKFATYGD--TAAS------GNDMTSKNFAKMTKECGVMDG-KTVTSTDVDILFS--KVKAK-----------NARNITYPEFME-ALKELSGKRFKGKSP----EEALQSIHKLIE-GKEPANVGTTKAVAAGGV-DRLTDTSKYTGSHKERFDES-GKGKGIAGRAD--------LAQN------TGYVGNYKGSGTY--DKTH---------

Cynops2 FS292922 ---MSAE----------LEAAFRKFAMHGN--TAAT------GNDMTGKNFSKLCKDTGVLNG-RDITSTDVDIVFS--KVKAK-----------AAKTINFAEFQQ-ALKELSVKRFKGKSA----DDALAETFKLVE-GREPVNAGVTKAQTVGGV-DRLTDTSKYTGMHKERFDES-GKGLGXXGXEN--------XXQX------XGYVG???????????????????????

Homo3 NP_776245 ---MAAST-----DMAGVKESFRKFAIHGD--PKAS------GQEMNGKNWAKLCKDCKVADG-KSVTGTDVDIVFS--KVKGK-----------SARVINYEEFKK-ALEELATKRFQGKSK----EEAFDAICQLVA-GKEPANVGVTKAKTGGAV-DRLTDTSRYTGSHKERFDES-GKGKGIAGRQD--------ILDD------SGYVSAYKNAGTY--DAKVKK-------

Gallus3 XP_003641947 ---MAGSA-----EMASLEESFRKFAIYGD--TKAT------GQEMNGKNWAKLCKDCKVIDG-KSVTGTDVDIVFS--KVKGK-----------TARVINYEEFKK-ALEELAPKRFKDKSK----EEAYEAICQLVA-GKEPINVGVTKAKNVGAV-ERLTDTSKYTGSHKERFDET-GKGKGKSGREN--------IVDN------SGYVSAYKNAGTY--DAKVKK-------

Anolis3 XP_003225414 ---MAES-----IDMASLEESFRKFAIYGD--TKAT------GQEMNGKNWAKLCKDCKVIDG-KGVTGTDVDIVFS--KVKGK-----------TARVINYEEFKN-ALEELAPKRFKDKNK----EEAYEAICKLVA-GKEPANVGVTKAKSVGAV-ERLTDTSKYTGSHKERFDES-GRGKGKSGREN--------IVDT------SGYVGAYKHAGTY--DAKVKK-------

Xenopus3 NP_001089831 ---MAENS-----DLTSLEESFRKFAIYGD--TKAT------GQEMTGKNWAKLCKDCKVIDG-KSVTGTDVDIVFS--KVKGK-----------SARVITCEEFKK-ALEELSGKRFKGKSK----EEAYEAICKLVV-GKEPVSAGITKPAATGAV-DRLTDTSKYTGSHKERFDES-GKGKGKGGRET--------IVEN------TGYVSSYKLAGTY--DAKVKK-------

Danio3 XP_687926 ---MAEST-----DMDQLLNSFKKFAVHGD--TKAT------GKELNGKNWAKLCKDCKVIDG-KNVTSTDVDIVFT--KVKAK-----------TSRVITYEEFQK-ALEELAPKRFKGQSK----EEALESIYKLIE-GKEPTNIGVTKVAKTAAV-DRLTDTSKYTGSHKERFDET-GKGKGKGGREE--------IVEH------TGYVGAYKNAGKY--DEKTKAK------

Tetraodon4 CAF95233 ---MTATTALL----GDVKIAFEKFAVHGD--TKAT------GKEMNGKNFAKICKESHIIDG-KNVNVTDVDIIFS--KVKAK-----------SARVITFEQFVH-ALAELAPKRYEGKSK----EEALLQIYSLLV-GKEPANMGITKVTKASAV-DRLTDTNKYTGTHKERFDES-GKGKGMAGRVD--------IPDM------SGYVAGYKSSGTY--EDKVKEA------

Suberites GH560390 ---MATG----------LESTFNGFCSFGASKDGAA--------LMDNAKFAKLFRDLKLLD--KKFTSTDVDIIFNRPEVKAK-----------GERKINFAQFQA-ALKLVAEKKYPGDS------DGLKKLTDKILTGKGPATSGATKFKSSGAV-DRLTDTSKYTGSHKERFDES-GKGKGLEGRDTGAKGHGMAAGSVAGQ---AGYVSGYKHEGTY--DKKK---------

Caenorhabditis NP_491219 ---MAAAAGFNWDD-ADVKKRWDAFTKFG----AAT------ATEMTGKNFDKWLKDAGVLDN-KAITGTMTGIAFS--KVTGP------------KKKATFDETKK-VLAFVAEDRARQSKK--PIQDELDAITEKLAKLEAPSVGGAAKANAAGVY-SRLTDHTKYTGAHKERFDAE-GKGKGKSGRA---------DTTEN-----TGYVGAYKNKDSY--DKTHGK-------

Drosophila NP_648881 PATELAQLALEDEPKVSFSDQFKAFSKFGD--SKSD------GKLITLSQSDKWMKQAKVID--KKITTTDTGIHFK--KFKAM--------------KISLSDYNK-FLDDLAKTKK----------VELSEIKQKLASCGAPGVVSVSAGKAAAAV-DRLTDTSKYTGSHKERFDAS-GKGKGIAGRRN--------VVDG------SGYVSGYQHKDTY--DNAH---------

Anopheles XP_308808 ETNGSATPANKPVCSAAFKEQFKAFSKFGD--TKSD------GKHLTLSQSDKWMKQAKVIDKKITTTDTGIHFKKL--KSMKL----------------TYEDYNK-FLDDLAKTKK----------VELDEIKNKMANCGAPGVHNATPGKAAETV-ARLTDTSKYTGSHKQRFDET-GKGKGIAGRKD--------MVDQ------SGYVSGYGHKNTY--DKTH---------

Daphnia EFX7974 ??????SPRPGSGAKNPLTELFRAFAKFGD--SKAD------GKAISLSQSDKWMKQAKVIDGKKITATDTGIYFKK--HKSLK---------------LGLADYQK-FLEELAKAKK----------VELTEIREKMIQCGPPGTTGTTVTMKTAAV-DRLTDSAKYTGSSRMRFDES-GRGRGMDGRRD--------KPDG------SGYVQGYDNKNSY--DKSH---------

Strongylocentrotus XP_782492 ---MSDG---------QLQDVFKSFCAFGAGSKDAA-------PVMDNSKWGKMFRDLKLYD--KKFTSTDTDIIFN--RPEVK---------SKTDRKINFAQFKK-ALELCAEKKY-------GSKDDVQKLIEKICAGKGPGTSGATKASKAGGV-DRLTDTSKYTGSHKERFDES-GKGKGLDGRKD--------FDAKAA----EGYVGGYKGKDTY--DKK----------

Amphimedon XP_003384590 ---MASA-------KTSLEDVFQSFCSFGEGLKGSA--------AMDNAKFAKLTRDVKILD--KKLTSTDVDIIFS--KVKAK-----------TDRKINFEQFKE-AVRLMADKKYPGD------PDGERKLIDKITAGSGPKVQGVTKTVDSPLL-ERMTDTSKYTGTHKERFDES-GKGKGLAGRDSFQKGAGMAPDGFSGN---ASYVHGYKHEGTY--DKKVKK-------

Nematostella XP_001628751 ---MSDD---------QLQAKFESFCAFGAGAKGAQ-------PLMDNAKFGKMFRDLHLYD--QKFTSTDTDIIFS--RTEVK---------PKTERKINFNQFKV-ALGLCAEKKF-------GSKDQVGKLTEKICKGKGPATSGATKAVKVGGV-ERLTDTKCYTGSHKERFDKS-GKGKGIEGRVD--------RDDKAA----QGYVGNYKGEGTY--DKTH---------

Hydra XP_002154495 -----------------MESVFKSFCAFGSRKDETV-------DLMDNAKFSKLARDLKILD--KKLTSTDVDIIFN--KVKSK-----------TERKITYHQFED-GVKLMAEKKY------PGDAEGYNKLKDLINSGSGPTASGVTKTAKSDTV-ERLTDTSKYTGSHKERFDES-GKGKGLDGRRE--------FDEKAS----AGYVGGYKEMNTY--DQNHK--------

Ciona XP_002124388 ---MGDK---------ELEAAYKKFMVMGN-SK---------ATKMTGKNFAKCLKDCKVLG--PKESTNSVDIIFS--TLKPN-----------SEKTIDFKQFKV-GLEKVAAEKK----------INKEDVFNKVINGGGPVMVGVTKTSKSGGV-EKMTDTSQYTGSHKERFGAD-GKGKGLDGRV---------DKVDA-----SGYVGNYKGDGTY--DQKVSK-------

Crassostrea CD647953 ---MASSGE-------DLDALVAKLKEFSL-TQTKN------ASKMDSKTVGKMAKECWPKPLQTRIDSS----VFP--KVMDK-----------TTKSINLDN-KE-QVKNFIKEAGIQYGDVTKKTGENHEKLLVDKVLAASLGIKKTAISKTGGL-HKMTDASQYTGAHKERFDAS-GKGKGAAGRT---------DKAEN-----TGYVGNYKGQGTF--DKK----------

Oryza CT849204 ---MASGGGS------NLHEIFEKYARFGKTEAQIKEAKG--GLRIETKNVQKLCKESGVLD--AKYPSQLLDNDIM--RVIGKLVTSHPQHYPKGTKTFEREGFET-LVHQIAESKK----------TDYNAIVAKMSSVSGPSLAGTTGVANKANV-DRMTDTSKYTGAHKERFGDD-GKGKGIDGRE---------NRTEN-----SGYVGNYKGANTY--DKAHK--------

Hordeum BM815954 ---MASGG---------LKEVFDKYSRFG-TESQLKEKD----IRIESKNVQKLMKDTGVVD--SKYTTQLLDNDIA--RVLGKLTSGG--TYAKGIKTFELNGFKQ-LVDQIAESKK----------TSADQIVQQLNSSGGPSLVGVTGTANKDIT-SRMTDTSQYTGAHKERFDDS-GHGKGKEGRT---------DAANN-----TGYVGNYKGSGTY--DXTH---------

Helobdella Helro1|135395 ---MARS----------LAEAFKQFAT----KADGKEQ--------TTADFTKWCKDAGVVG--KNCNSNHIDISFS--KAKAK-----------GARNITFENLD--ALITEMAKKYKDDVKM-GEAEAKEDLINKL-SGAKKLMHGVTGTMKVGGV-DRMTDTTLYTGAHKERFDEE-GKGKGLAGRS---------DVVDN-----TGYVGGYKNKDTY--DKKEK--------

Schistosoma AAX27359 ---MSSD---------ELKQSFLSFCNFVKKGSTTA----------TDKTIKKICTDCQIYS--KKLDANRIDIEFR--GHIGS-----------TKRDVDFPGFVS-FLEGRLAKVYAAANG--MEQEEAVIELKRKIAEASPAIHGGTKISSDPTT-SRLTDVKTFTGSHKERFDAQTGKGLGKAGRVDPKTIFYNKWNFNSKKIKNSFI-------------------------

Cs GAA47940 ---MASSNA-------DLKKAFLAYCSVIDHESTKG----------NSSCVRKMCTDSGINK--TKMKQNDIDLEYV--RCFGT-----------SKEGIDFKAFLV-FVEEYLGPVYGRMNG--MEKEDAIAEVKRRLSSVSPQL-------------------------------------------------------------------------------------------

Batrachochytrium BDEG_06075 ---MAT--------VEDLYMTFGSFCQFGSSRNLSGSMTDISGPTMDGSKWAKFCRDTGIID--KHITTTDIDIWFN--KVKAK-----------TVRKIDFEQFQA-ALHLVAAKRYGSSK--PPTEAYNLLVRSILNSGARPVATG-TITTSDSVT-QRLTDHTHYTGTHKNRFDEA-GQGLGLAGRDT--------HSRT-------NELSKIVNRKEA--DIRGVPLAASPIG

Cyanophora EC665897 ---------MPIIGDADLKNIFVTFASFGAGNAGAT--------TMDNARFAKFCKDTKIVD--SKYTTTSIDIIFN--KVKKV----------KTERRINFEEFKH-ALEEIGKEKYKGA------ADGVEKVHGLISAHGGPSNSG-TQADASGIY-SKLTDHTTYTGVYKERFDED-GKGKGVV-----------------------QIN-------------GGAGK------

Is XP_002404704 ATPPGSPAPSSPVPPTCFEGQFKAFAKFGDSKST--------GDAITLSNSDKWFKQAKVIDG-KKITTTDTGIYFK--QVAK----------------------------------------------------------------------------------------------------------------------------------------------------------

Mo XP_003742023 VTPPESPEPEEPVG-EDFGEMFKSFARVGDSKGA--------GDLITLSNSDRWWKQAKVIDG-RRISTTDTGIYFR--KIAK------------TKRTLTFREYQL-FVEGIAKSKK------IPVEEIRFKLCN----CGPPTVGGRFQLDDAFGARKVRMRE------------------------------------------------------------------------

Dap EHJ66593 ---------MGEEEPASLDGQFYEFAKMMDK----KRD----GTTITLYNSDFWFRQCKILD-DRKVTMTDTGILFN--KFGK--------------SEINWDEWNE-FLVDLCELKG------LDLEKAQDTLTN----CGLPGQTP-VVVPQYRDFFLTYKPKEKMLF-------------------------------------------------------------------

Dm N P_648370 -----MADDEDKPKKHTLDSLFLVYSNFQVIPTDIENE---YFDSILLSQLDAWLEQAKLMP--NPITRTQTGLIYM--RYKKW-------------R-LEYEDFLE-VLNNLASDNN------LAIDEMKQIMID----AGVPNGADVVIVVK-----------------------------------------------------------------------------------

Dse XP_002029959 -----MADDEDKPKKHTLDSLFLVYSNFQVIPTDIENE---YFDSILLSQLDAWLEQAKLMP--NPITRTQTGLIYM--RYKKW-------------R-LEYEDFLE-VLNNLASDNN------LPIDDMKQIMID----AGVPNGADVVIVVK-----------------------------------------------------------------------------------

Dy XP_002094265 -----MADDEDKPKKHTLDSLFMVYANFQVIPTDIENE---VFDSILLSQLDAWLEQAKLMP--NPITRTQTGLIYM--RYKKW-------------R-LEYEDFLE-VLNNLASDNN------LQIEDMKQIMVE----AGVPNGADVVIVVK-----------------------------------------------------------------------------------

Dw XP_002062203 -----MADEDDKPKKYTLDSLFMVYCNYKLVPTELENE---EFDCILLSQIDAWLEQAKLMP--MPITRTQTGLIYM--RYKKW-------------R-LEFEDFLE-VLQQLATDNE------LNYDELKQTLID----AGVPSGATEIVTVK-----------------------------------------------------------------------------------

Dpp XP_001353716 -----MADDDDKPKKHTLDTLFIVYCNYKVISTELENE---EFDCILLSQIDAWIEQAKLMP--NPITRTQTGLLYM--RYKKW-------------R-LEFEDFVE-LLTQLASEND------LVFDEFKQLLVE----AGPPTGATDIVMVK-----------------------------------------------------------------------------------

Dv XP_002047114 -----MADDEDKPKKHTLDSLFHVYCNHKVIGNELENE---EFHSILLSQLDNWLQQAKLMP--VPITRTQTGLLYM--RYKKW-------------R-LDYEDFLE-VLQHLSTDAD------LNYEDFKVTLVA----AGPPTGATEIVVVK-----------------------------------------------------------------------------------

Dg XP_001983728 -----MADDEDKPKKHTLDTLFQVYCNYKLAAAELETE---EFHSILLSQLDSWLTQARLMP--APITRIQTGLIYM--RYKKW-------------R-LEYEDFLE-VLQILCSESD------LNYDQLKETLIA----AGPPLGATEVVVVK-----------------------------------------------------------------------------------

Cq XP_001862283 ----MAKVIVPGPELPSLESMFSSYAKYRPSLNTFQGD----GKRILLSQSDAWMQQARLVGAKRVFSLTETGVMFF--KLSKS-----------T---LDFDEFLQ-FLESLCASKG------VGFEEVKTSLVS----CGPPGIVS-----------------------------------------------------------------------------------------

Ag XP_556944 SNPPTTASKVKPP---TLPSMFTLFAKYRPTLNSFQGD----GKRILLSQSDCWMQQANLIGP-KHFTLTQTGLIFF--EFRKS-----------T---LDYDEYLQ-FLALLCNEKQ------VSVEEVKEKLTN----CGPPGITS-----------------------------------------------------------------------------------------

Tc EFA09619 -----------MSGCGNIEHQFKAVT-HSNS-------------KITIDQINQWFTDAKL-N--KKITPEDTKLCFE--KFKAE-----------T---IDLTSFVK-FLQDLSEQKK------LPVGELEEKLAS----CELLTGGGTVANNTVGKKITDAMQSVVEDGHKPSKRTCKRMK-------------------------------------------------------

Si EFZ11240 ?????????LAALNRVSFLASFKAFFKFGDS----KSD----GKLITLSQSDKWMKQAKVIDG-KKITTTDTGIYFK--KQKLN-----------SVNCIC--AHLS-IVHVTCIICA------HM---------------------------------------------------------------------------------------------------------------

Cf EFN74475 ????????????????????????????????????????MGVSLIPLSQSNKWLMSAGILD--MKLTTTDTGLAFF--KFRK--------------RALSYVEYLT-YLKDLATSYN------LNFEDMKYRMQI----CGKPSIMREDIKT------------------------------------------------------------------------------------

Thp XP_766449 ---------------MKLSELFERYRDQN----------------LKGRMFVKMFRDAGLITS---YD-NSLDLIFA--KYKSK-----------CSG-INYEQFLK-SLEEVSRL-------LDMKVPELKQRLRES---EGPIYRG-TEPLAVRLH-D---DKRLYTGVHLHGGP---------------------KIGKQ----------------------------------

Tha XP_953847 ---------------MEISKVFDNYKDQNN--------------VLKGRMFIKMFKDANLIS--SSSETNNLDLIFT--KYKSK-----------FSG-INYDQFLK-SLKDVSKV-------LNMEPSELELKLKRT---NGPIYKG-TETQPVR-LYD---DKSLYTGVHLHGGP---------------------RIIDK----------------------------------

Bb XP_001610770 ------------MASSELHQIYQHYINKST-------------GQLEGRMFVKIFKQANLLD--QKLNTNDLDIIFV--KHRTK-----------GSRTMDFSGFEK-AIQAAAVA-------LGIDYQEIVERVLKA---GAPVYAG-TETLPVR-FYD---DKNSYTGVHAHGGP---------------------SVK------------------------------------

ETH_00015030 -MHQVFLVLFGKFKKMSLEEAFRVYTKGAA--------------EMDGRTFGKMLKDCGVLSS-SKMTAVDADLIFA--KVKDK-----------GSKKISFLQFQE-ALKLVAAK-------KGMDLQTLQQKLAAEG-SEGPILTG-TKADNVR-FHD---DKSTYTGVHKMGGP---------------------TTVDDGRV-QFNDLSKFCDRSEY---DIRGVKKGIIENK

Nc CBZ53835 --------------MATASGAFQVYTKGSG--------------DMDGRTFVKILKDTAILDG-KTLTTVDADLIFT--KVKAK-----------GAKKIDYAQFEE-ALKLVGEK-------KKVSTEQIVSKLASG--ETGPILTG-TKADNVR-FHD---DKNTYTGVHKHGGP---------------------TLVDEGRT-QFSDLSNICDRSDY---DVRGVKKGVAE--

Py XP_726013 -----------------METVFNIYTKNMP--------------DMDSRTFVKILKDSKLLN--KKITAVDADITFA--RVKTQ-----------GSKRIKYDQFVE-AIKYITEK-------NKLDYDQFVEQLCNEA-SNGPILYG-TKAEATR-FHD---DKSTYTGVHKLGGP---------------------TTIDKNKT-HFSNISEITDRSEC---NIRGVNLSVEKNI

Pb XP_674367 -----------------METVFNIYTKNMP--------------DMDSRTFVKILKDSELLN--KKITAVDADITFA--KVKTQ-----------GSKRIKYDQFVE-AIKYITEK-------NKLDYDQFVEKLCNEA-SNGPILYG-TKAEATR-FHD---DKSTYTGVHKLGGP---------------------TTIDKNKT-QFSSISEITDRSEC---NIRGVNLSVEKNV

Pc hXP_740774 -----------------MEGVFNIYTKNMP--------------DMDSRTFVKILKDSKLLS--KKITAVDADLAFA--KVKTK-----------GSKRIKYDQFVE-AIKHLTEK-------YKLDYDQFVGKLCNEA-SNGPILYG-TKAEATR-FHD---DKSTYTGVHKLGGP---------------------TTVDKNRT-QFSDISEITDRSEC---NIRGVNISVEKNM

Pv XP_001613902 -----------------MENAFYIYTKNEA--------------DMDSRTFVKILKDAKLLS--KKLTAVDADLTFA--KVKAK-----------GAKRINYDQFVE-AVKHLVDK-------HKLDYDQFVEKLCNEA-SSGPILYG-TKAANVR-FHD---DKSTYTGVHKMGGP---------------------TTVDKNKT-HFSDISEITDRSEC---NIRGVNLSVEKNL

Pk XP_002262452 -----------------MENAFYIYTKNEA--------------DMDSRTFVKILKDSKLLN--KKLTAVDADLTFA--KVKAK-----------GAKRINYDQFVE-AVKHLVDK-------HKLDYEKFVETLCNEA-SSGPVLYG-TKAANVR-FHD---DKSTYTGVHKLGGP---------------------TIIDKNKT-HFSDISEITDRSEC---NIRGVNINVEKNL

Pf XP_001350760 -----------------MENAFYVYTKNLP--------------DMDSRTFVKILKDAKLLN--KKFTTVDADLIFA--KVKSK-----------GAKRINYDQFLE-AVKCIVEK-------NKLNYDKFVETLCQEA-SKGPILYG-TKTENVR-FFD---DKSTFTGVHKQGGP---------------------SIIDKNKT-QFSDLSEITDRSEY---DIRGVKMDVAKNV

Tg XP_002369913 --------------MSGLDAVFKSFTHDAP--------------AMDGRTFVKLCKDCKAFD--KNYTTTDADLIFT--KVKAK-----------GAKTITFAEFEA-AIDLIAEK-------KKVSAQELAAQISS---ASGPVYSG-TKALPNK-FHD---DKSLYTGVHANGGP---------------------STVDGNVN----DISQILDRSAA---TVRGTKM------

Tth XP_001023601 -------------MQASLEGVFKKFTANKA--------------DMDGKTFAKFAKDCGLLD--KKLTATDIDLIFA--KVKTSS----------AVRTITFAQFEK-GLDQMATK-------KGISLDALKEKVTS---AGGPTFTG-TKADAVK-FHD---DKSLYTGVYANGGP---------------------STVDIG-NGKISDISQLCDRTGA---DVRGVKK------

Pt XP_001436768 -------------MQGNVQQVFLQFTANKP--------------EMDGKTFAKVSKDCHLLD--KKLTSTDVDLIFA--KIKPTP----------AARSITYAQFEK-GLQMMAEK-------KGVGVQDVHNQILN---AGGPHFQG-TKADAVK-FHD---DKNLYTGVHANGGP---------------------STIDKN-HG---GLNTICDRSQA---DVRGSQKMMKNI-

Os CT850609 ----MEPAATTTEVETTLDQVFKNFNAGGL--------------EMDNRQFAKVAKDTGILD--KKLTATDVDLIFN--KVKANP----------AIRKIKYSQFEE-AMTHFATK-------KGIKEDALIEMILK---KGGPKFTA-TKADFVK-FHD---DKNTYTGVHTKGGP---------------------TTVDNK-----ITLSNLADRSKA---DVRGVKISK----

Tae CD868723 -----------MEAATTLESVFKAF-AGGA--------------EMDGRAFVKLTKDTGLLD--KKLTTTDVDLIFA--KIIDK-----------TSKKANFTAFKS-GVVQFAAK-------KGISEADCTALIVK---AGGPKYEG-TKADFVK-FHD---DKSTYTGVYAKGGP---------------------TNVDAGRGGQVSDISQTCDRTSA---DVRGIKK------

Pam EW705544 ????????????????????????SHYGRG--------------HLIDKNLTLAIVDLVFNQ--IKPKGGRTITFKV--FCEGLD----------KLGSYKYP-----AEFKQGGA-------AATTPKLVELINKQKV----PVSTG-TKAQANK-FHD---DKKLYTGVHAKGGP---------------------STNDNR-----ITLSGLANRAPA---NARGLNK------

Tb XP_844424 --------------MEAVFYAFASFGTAP-------------TKEMDNAHFSKMLKEAKIIG--KTFTSTDADLLFN--KIKAK-----------GARKITFTEFNTRALPDIATK-------LKMTPEQVAEILTK----ASPASNS-TKAEAVK-FHD---DKNLYTGVYKAGGP---------------------TNVDRN----AGSLSGVVDRRVD-QVDVRGTTSSQK---

Tc XP_806144 ------------MSIESAFYAFASFGGAP-------------TKEMDNAHFSKMLKETKVIG--KQFTSTDADLLFN--KVKAK-----------GARKITLSDFVDKAVPEIASK-------LKKSAEELIADISS----CSPEARA-TKADAVK-FHD---DKNMYTGVYKAGGP---------------------TNVDRN----SGSLSGVVDRRVA-QTDVRGTTASQK---

Lm XP_001686248 -----------MDNFQATFEAFASFGSAP-------------SKEMDNSHFSKMLKECKIIG--KSFTSTDADLLFS--KVKAK-----------EARKISFTEFKEKAIPEIAAK-------MKKTPADIEAMIAN----AAPKSSG-TKADTVR-FHD---DKSTYTGAAKQGGP---------------------TNVDRN----AGSLAGVVDRRQE-TIDNRGTTAKQI---

Li XP_001468533 -----------MDNFQATFEAFASFGSAP-------------SKEMDNSHFSKMLKECKIIG--KSFTSTDADLLFS--KVKAK-----------EARKISFTEFKEKAIPEIAAK-------MKKTPADIEVMIAN----AAPKSSG-TKADTVR-FHD---DKSTYTGAAKQGGP---------------------TNVDRN----AGSLAGVVDRRQE-TIDNRGTTAKQI---

Lb XP_001563404 FYFFRSRINYLIEDPETIFEAFASFGSGP-------------SKEMDNSHFSKMLKECKIIG--KTFTSTDADLLFS--KVKAK-----------EARRISFTEFKEKAIPEVAAK-------MKKTPEEIETMITN----AAPKSNS-TKADAVR-FHD---DKSTYTGAAKQGGP---------------------TNVDRN----AGSLAGVVDRRQP-TLCGCVIMLYEPRSG

Al EC630993 ???????????????????????????????????????????????????????RDTKLLD--KKFTATDADLIFS--KAKAK-----------GERKINFATFRDKAVPLIAEK-------KGCSVDAVVATACG---GGGPSSSG-TKADAVK-FHD---DKSLYTGVYARGGP---------------------TNVDKD----KITLAGVVSDHDALTCDVRGIVRK-----

Dp EC843061 ?????????????????????????????????????????????MDNSRFNKFCKDAGIIDG-RKFTSTDADLIFT--KSKGK-----------GERRISYSVFASSTLEEVAKK-------KAISKDQLVAQITAK--SSAPSSSG-TQAENVA-LHD---DKSLYTGVYSKGGP---------------------TSNDL---G-TSDLSFVTNREDA---DVRGVQI------

Chr XP_001695016 --MSD--------ALKNAFIAFASYGKGQMM-----------KQDMDNKNFSKCIKDSGILD--KVITSTEVDITFM--KVKAK-----------TDRTINFAQFCT-ALEHFAAK-------RGVSVDSLHAKVEA----ASPTSNA-TQAEAVK-FHD---DKNLYTGVYKNGGP---------------------TNIDKQ--A-AGGLAGHLDRSPA---DVRGVKF------

Vc XP_002946668 --MAD--------ALKEAFIAFASYGKGQEI-----------KQDMDNKNFSKCMKDSKIIDG-KCITNTEVDITFM--KVKAK-----------TDRTINYAQFCA-ALDHFAQK-------KGCTQAELAQKVAE----ASPTSNA-TKAQAVK-YYD---DKSMFTGVHKNGGP---------------------TTVDK--M-RAGGLANLCDRSPA---DNRGVKY------

Chv EFN56830 --MVSRATVNRSNDLRVVFTAFAAFGSGRDI-----------SSGLEGRAFSKIFKDAGLYC--RKFTVTDADLIFT--SVKPK-----------GGKRISYDAFEQ-ALQKVATK-------KGVSMAEVVSTIVA---AGGPKSNG-TRAEACR-FYD---DKSNWTSTARNGGP---------------------TNVDGQ-----KDLSSLCDRTAA---DARGISAKSNFSR

Mp ACO68100 ---------------------------------------------MESRGFIKMLRDCNVLN--KRFNDAAADIIFT--KVKDR-----------GERFIDIHDFAL-ALHFVAEE-------KGTTYETLVAQICERVATHAPDAHG-TKAAFTR-LHD---DKDTYTGAY-----------------------------------------------------------------

Pem EER04926 --------------------MYKAFTGGDN--------------MMDGRQFAKLCKDCQIVE-KGSLSVNDIDIIFA--KVRSR-----------GERKIEFGQFME-ALQEVADR-------LDKPISWAKEKVVE---CDGPTIRT-SVGPSAR-PSI---DAGSDRKVWRNGGP---------------------QTVDTGASSQAPA-TEVTKRASG---STSTPAGK-----
