# Supplementary material for: A New Protein Superfamily: TPPP-Like Proteins
Source: PLoS One. 2012 Nov 14;7(11):e49276. doi: 10.1371/journal.pone.0049276 (PMC3498115; doi:10.1371/journal.pone.0049276)
Supplement: Figure S2 — Multiple sequence alignments of TPPP-like proteins by ClustalW used for constructing the phylogenetic trees. (DOC) [file pone.0049276.s002.doc]

CLUSTAL 2.1 multiple sequence alignment – short type TPPPs (Supplementary Fig. 3)

Thp XP_766449 ---------------MKLSELFERYRDQN-----LKGRMFVKMFRDAGLIT-SYD--NSLDLIFAKYKSKCSG--INY

Tha XP_953847 ---------------MEISKVFDNYKDQNN---VLKGRMFIKMFKDANLIS-SSSETNNLDLIFTKYKSKFSG--INY

Bb XP_001610770 ------------MASSELHQIYQHYINKST--GQLEGRMFVKIFKQANLLD-QKLNTNDLDIIFVKHRTKGSRT-MDF

ETH_00015030 -MHQVFLVLFGKFKKMSLEEAFRVYTKGAA---EMDGRTFGKMLKDCGVLSSSKMTAVDADLIFAKVKDKGS-KKISF

ETH_00003465 GGPWGPQLLQPRSAMATLEELFSNFGRGK----DLDSRTFVKLCKDCKLLG-GGLTQTDCDLIFTKCKAKGA-ARLTF

Nc CBZ53835 --------------MATASGAFQVYTKGSG---DMDGRTFVKILKDTAILDGKTLTTVDADLIFTKVKAKGA-KKIDY

Nc CBZ50502 --------------MSGLDGVFKSFTHDAP---AMDGRTFVKLCKDCKVFD-KNYTTTDADLVFTKVKAKGA-KTITF

Nc CBZ53276 ---------------MSLASVFQSYTQGKG---DMDSRTLVKLCKETGMID-KQTTATDIDLIFTKCKARGA-KRLTA

Py XP_726013 -----------------METVFNIYTKNMP---DMDSRTFVKILKDSKLLN-KKITAVDADITFARVKTQGS-KRIKY

Pb XP_674367 -----------------METVFNIYTKNMP---DMDSRTFVKILKDSELLN-KKITAVDADITFAKVKTQGS-KRIKY

Pch XP_740774 -----------------MEGVFNIYTKNMP---DMDSRTFVKILKDSKLLS-KKITAVDADLAFAKVKTKGS-KRIKY

Pv XP_001613902 -----------------MENAFYIYTKNEA---DMDSRTFVKILKDAKLLS-KKLTAVDADLTFAKVKAKGA-KRINY

Pk XP_002262452 -----------------MENAFYIYTKNEA---DMDSRTFVKILKDSKLLN-KKLTAVDADLTFAKVKAKGA-KRINY

Pf XP_001350760 -----------------MENAFYVYTKNLP---DMDSRTFVKILKDAKLLN-KKFTTVDADLIFAKVKSKGA-KRINY

Tg XP_002369913 --------------MSGLDAVFKSFTHDAP---AMDGRTFVKLCKDCKAFD-KNYTTTDADLIFTKVKAKGA-KTITF

Tg XP_002367917 ---------------MSIAGVFQSYTQGKG---DMDSRTLVKLCKETGVID-KQTTPTDIDLIFTKCKAKGA-KRLTC

Tth XP_001023601 -------------MQASLEGVFKKFTANKA---DMDGKTFAKFAKDCGLLD-KKLTATDIDLIFAKVKTSSAVRTITF

Tth XP_001023599 -------------MNSSLEGVFKKFTGGKI---EMDGKTFAKFAKDTGLLD-KKLTATDVDLIFAKVKGSSAIRCINI

Pt XP_001436768 -------------MQGNVQQVFLQFTANKP---EMDGKTFAKVSKDCHLLD-KKLTSTDVDLIFAKIKPTPAARSITY

Pt XP_001423901 -------------MQGTVQQVFLQFTANKP---EMDGKTFAKVSKDCHLLD-KKLTSTDVDLIFAKIKPTPAARSITY

Os CT850609* ----MEPAATTTEVETTLDQVFKNFNAGGL---EMDNRQFAKVAKDTGILD-KKLTATDVDLIFNKVKANPAIRKIKY

Tae CD868723* -----------MEAATTLESVFKAF-AGGA---EMDGRAFVKLTKDTGLLD-KKLTTTDVDLIFAKIIDKTS-KKANF

Tth XP_977236 ---------------MSLLSAFKKFTNDRS---TMEVKIFIGALTDSGVFN-GRVNPSQAEYIFDKVKNSANLRGINY

Tth AAL79503 ??????????????????????????GDRS---TMEVKIFIGALTDSGVFN-GRVNPSQAEYIFDKVKNSANLRGINY

Tth XP_977237 ---------------MSLSAAFKKFTNQRS---TMDSKTFVNTLADSGIFN-FKITTHQSEQIFEKVKNSPNLRGINY

Tth XP_977235 ---------------MSLAAAFKKFTNEKS---SMDVKIYLSVLKESGVLN-YKVQADLAENFFNTIKNNPNLRGISY

Pam EW705544* ????????????????????????SHYGRG---HLIDKNLTLAIVDLVFNQ-IKPKGGRTITFKVFCEGLDKLGSYKY

Tb XP_844424 --------------MEAVFYAFASFGTAP--TKEMDNAHFSKMLKEAKIIG-KTFTSTDADLLFNKIKAKGA-RKITF

Tc XP_806144 ------------MSIESAFYAFASFGGAP--TKEMDNAHFSKMLKETKVIG-KQFTSTDADLLFNKVKAKGA-RKITL

Lm XP_001686248 -----------MDNFQATFEAFASFGSAP--SKEMDNSHFSKMLKECKIIG-KSFTSTDADLLFSKVKAKEA-RKISF

Lm XP_001686249 ----------------------------------MDNSHFSKMLKECKIIG-KSFTSTDADLLFSKVKAKEA-RKISF

Li XP_001468533 -----------MDNFQATFEAFASFGSAP--SKEMDNSHFSKMLKECKIIG-KSFTSTDADLLFSKVKAKEA-RKISF

Li XP_001468534 ----------------------------------MDNSHFSKMLKECKIIG-KSFTSTDADLLFSKVKAKEA-RKISF

Lb XP_001563404 FYFFRSRINYLIEDPETIFEAFASFGSGP--SKEMDNSHFSKMLKECKIIG-KTFTSTDADLLFSKVKAKEA-RRISF

Lb XP_001563405 -----------MSNFQATFEAFASFGSGP--SKEMDNSHFSKMLKECKIIG-KTFTSTDADLLFSKVKAKEA-RRISF

Al EC630993* --------------------------------------------RDTKLLD-KKFTATDADLIFSKAKAKGE-RKINF

Dp EC843061* ----------------------------------MDNSRFNKFCKDAGIIDGRKFTSTDADLIFTKSKGKGE-RRISY

Chr XP_001695016 --MSD--------ALKNAFIAFASYGKGQMMKQDMDNKNFSKCIKDSGILD-KVITSTEVDITFMKVKAKTD-RTINF

Chr XP_001695015 --MSDPFMCSRGEALRLAFIAFASYGKGQELKQDMDNKNFSKCIKDSGIMDAKCITATEVDITFMKVKEKTA-RTINF

Chr XP_001701105 ---------MSEDLAQHVFIKYASIGKTGIANATLDGSKFAKLCRESKLIG-GGLTPMDVEGIFLRVSKSFG-R-INY

Vc XP_002946668 --MAD--------ALKEAFIAFASYGKGQEIKQDMDNKNFSKCMKDSKIIDGKCITNTEVDITFMKVKAKTD-RTINY

Chv EFN56830 --MVSRATVNRSNDLRVVFTAFAAFGSGRDISSGLEGRAFSKIFKDAGLY-CRKFTVTDADLIFTSVKPKGG-KRISY

Mp ACO68100 ----------------------------------MESRGFIKMLRDCNVLN-KRFNDAAADIIFTKVKDRGE-RFIDI

Pem EER04926 --------------------MYKAFTGGDN---MMDGRQFAKLCKDCQIVEKGSLSVNDIDIIFAKVRSRGE-RKIEF

Pem EEQ99821 ------------MATANLLEMYKAFTGGDT---MMDGRQFAKLCRDCNIVDKKGLSVNDTDIVFAKVRSRGE-RKINF

Thp XP_766449 EQFLK-SLEEVSRLLDMKVPELKQRLRES---EGPIYRGTEPLAVRLHDDKRLYTGVHLHGGPKIGKQ----------------------------------

Tha XP_953847 DQFLK-SLKDVSKVLNMEPSELELKLKRT---NGPIYKGTETQPVRLYDDKSLYTGVHLHGGPRIIDK----------------------------------

Bb XP_001610770 SGFEK-AIQAAAVALGIDYQEIVERVLKA---GAPVYAGTETLPVRFYDDKNSYTGVHAHGGPSVK------------------------------------

ETH_00015030 LQFQE-ALKLVAAKKGMDLQTLQQKLAAEG-SEGPILTGTKADNVRFHDDKSTYTGVHKMGGPTTVDDGRV-QFNDLSKFCDRSEY---DIRGVKKGIIENK

ETH_00003465 SEFEA-AMEAVAAKKKCSLEELLQQVGP---GGGPQFRGTAAEAVRFYDDKSTFTGVHAHGGPSTVDKKGKGTFITLADICDRSTP---DVRGINKNFK---

Nc CBZ53835 AQFEE-ALKLVGEKKKVSTEQIVSKLASG--ETGPILTGTKADNVRFHDDKNTYTGVHKHGGPTLVDEGRT-QFSDLSNICDRSDY---DVRGVKKGVAE--

Nc CBZ50502 AEFEA-AIGLIAEKKKVSAQELSAQISS---ASGPVYSGTKALPNKFHDDKSLYTGVHANGGPSTVDGNIN----DISQILDRSAA---TVRGTKM------

Nc CBZ53276 DDFEK-VVEEIAARKKKPVDEIIQQLCS---SAGPSFSGTKTDAVRFYDDKTTFTGVHAHGGPSTVDTPSTKFNITLADICDRSTP---DIRGVNKNFQKS-

Py XP_726013 DQFVE-AIKYITEKNKLDYDQFVEQLCNEA-SNGPILYGTKAEATRFHDDKSTYTGVHKLGGPTTIDKNKT-HFSNISEITDRSEC---NIRGVNLSVEKNI

Pb XP_674367 DQFVE-AIKYITEKNKLDYDQFVEKLCNEA-SNGPILYGTKAEATRFHDDKSTYTGVHKLGGPTTIDKNKT-QFSSISEITDRSEC---NIRGVNLSVEKNV

Pch XP_740774 DQFVE-AIKHLTEKYKLDYDQFVGKLCNEA-SNGPILYGTKAEATRFHDDKSTYTGVHKLGGPTTVDKNRT-QFSDISEITDRSEC---NIRGVNISVEKNM

Pv XP_001613902 DQFVE-AVKHLVDKHKLDYDQFVEKLCNEA-SSGPILYGTKAANVRFHDDKSTYTGVHKMGGPTTVDKNKT-HFSDISEITDRSEC---NIRGVNLSVEKNL

Pk XP_002262452 DQFVE-AVKHLVDKHKLDYEKFVETLCNEA-SSGPVLYGTKAANVRFHDDKSTYTGVHKLGGPTIIDKNKT-HFSDISEITDRSEC---NIRGVNINVEKNL

Pf XP_001350760 DQFLE-AVKCIVEKNKLNYDKFVETLCQEA-SKGPILYGTKTENVRFFDDKSTFTGVHKQGGPSIIDKNKT-QFSDLSEITDRSEY---DIRGVKMDVAKNV

Tg XP_002369913 AEFEA-AIDLIAEKKKVSAQELAAQISS---ASGPVYSGTKALPNKFHDDKSLYTGVHANGGPSTVDGNVN----DISQILDRSAA---TVRGTKM------

Tg XP_002367917 EDFEK-AVEEIAKRKNKSVDEITQQLCS---SSGPSFSGTKADAVRLHDDKSTFTGVHAHGGPSTVDTPSRGQAISLADICDRSTP---DIRGVNKNFQKS-

Tth XP_001023601 AQFEK-GLDQMATKKGISLDALKEKVTS---AGGPTFTGTKADAVKFHDDKSLYTGVYANGGPSTVDIG-NGKISDISQLCDRTGA---DVRGVKK------

Tth XP_001023599 RQFEE-GINQFAAKKGISAQDLREKVTA---SNGPSYSGTKADAVRFHDDKSLYTGVYANGGPSTIDIG-SGKISDISQLCDRTSA---DVRGVKH------

Pt XP_001436768 AQFEK-GLQMMAEKKGVGVQDVHNQILN---AGGPHFQGTKADAVKFHDDKNLYTGVHANGGPSTIDKN-HG---GLNTICDRSQA---DVRGSQKMMKNI-

Pt XP_001423901 AQFEK-GLQMMAEKKGVGIQDVQNQILN---AGGPHFQGTKADAVKFHDDKNLYTGVHANGGPSTIDKN-HG---GLNTICDRSQA---DVRGVKK------

Os CT850609* SQFEE-AMTHFATKKGIKEDALIEMILK---KGGPKFTATKADFVKFHDDKNTYTGVHTKGGPTTVDNK-----ITLSNLADRSKA---DVRGVKISK----

Tae CD868723* TAFKS-GVVQFAAKKGISEADCTALIVK---AGGPKYEGTKADFVKFHDDKSTYTGVYAKGGPTNVDAGRGGQVSDISQTCDRTSA---DVRGIKK------

Tth XP_977236 QQFET-CLSQIAAQGGMTRQQIEGMLIEQS----KKLGAKEGDPSRFFYDKSSYTGVHTKGGPSTIDNN--G-YSHLSQLCDRSGA---NVRGVNK------

Tth AAL79503 QQFET-CLSQIAAQGGMTRQQIEGMLIEQS----KKLGAKEGDPSRFFYDKSSYTGVHTKGGPSTIDNN--G-YSHLSQLCDRSGA---NVRGVNK------

Tth XP_977237 QQFET-CLSLVASQTGCTRQQIEGLVLEYS----KKFTTKQADPSRFFYDKSTYTGVHTKGGPSTLDNN--G-YSQLSQLCDRSSA---NVRGIKN------

Tth XP_977235 PQFEQ-CLSQIALKNAQTKQLVEGMVLEYS----KKLGAKKSDPNRFFYDKSTYTGVHNNGGPSTIDNN--G-ISHLSQLCDRSGA---NVRGVKF------

Pam EW705544* P-----AEFKQGGAAATTPKLVELINKQKV----PVSTGTKAQANKFHDDKKLYTGVHAKGGPSTNDNR-----ITLSGLANRAPA---NARGLNK------

Tb XP_844424 TEFNTRALPDIATKLKMTPEQVAEILTK----ASPASNSTKAEAVKFHDDKNLYTGVYKAGGPTNVDRN----AGSLSGVVDRRVD-QVDVRGTTSSQK---

Tc XP_806144 SDFVDKAVPEIASKLKKSAEELIADISS----CSPEARATKADAVKFHDDKNMYTGVYKAGGPTNVDRN----SGSLSGVVDRRVA-QTDVRGTTASQK---

Lm XP_001686248 TEFKEKAIPEIAAKMKKTPADIEAMIAN----AAPKSSGTKADTVRFHDDKSTYTGAAKQGGPTNVDRN----AGSLAGVVDRRQE-TIDNRGTTAKQI---

Lm XP_001686249 TEFKEKAIPEIAAKMKKTPADIEAMIAN----AAPKSSGTKADTVRFHDDKSTYTGAAKQGGPTNVDRN----AGSLAGVVDRRQE-TINNRGFTA------

Li XP_001468533 TEFKEKAIPEIAAKMKKTPADIEVMIAN----AAPKSSGTKADTVRFHDDKSTYTGAAKQGGPTNVDRN----AGSLAGVVDRRQE-TIDNRGTTAKQI---

Li XP_001468534 TEFKEKAIPEIAAKMKKTPADIEVMIAN----AAPKSSGTKADTVRFHDDKSTYTGAAKQGGPTNVDRN----AGSLAGVVDRRQE-TIDNRGTTAYKNVKH

Lb XP_001563404 TEFKEKAIPEVAAKMKKTPEEIETMITN----AAPKSNSTKADAVRFHDDKSTYTGAAKQGGPTNVDRN----AGSLAGVVDRRQP-TLCGCVIMLYEPRSG

Lb XP_001563405 TEFKEKAIPEVAAKMKKTPEEIETMITN----AAPKSNSTKADAVRFHDDKSTYTGAAKQGGPTNVDRN----AGSLAGVVDRRQQ-TIDNRGTTAKQL---

Al EC630993* ATFRDKAVPLIAEKKGCSVDAVVATACG---GGGPSSSGTKADAVKFHDDKSLYTGVYARGGPTNVDKD----KITLAGVVSDHDALTCDVRGIVRK-----

Dp EC843061* SVFASSTLEEVAKKKAISKDQLVAQITAK--SSAPSSSGTQAENVALHDDKSLYTGVYSKGGPTSNDL---G-TSDLSFVTNREDA---DVRGVQI------

Chr XP_001695016 AQFCT-ALEHFAAKRGVSVDSLHAKVEA----ASPTSNATQAEAVKFHDDKNLYTGVYKNGGPTNIDKQ--A-AGGLAGHLDRSPA---DVRGVKF------

Chr XP_001695015 EQFAL-ALESFASKKGCPVAQLEEKIEG----AQPKNNATIAQAAQYHDDKSLYTGVYKNGGPTNVDKGPT-KAGGLASHLDRSPA---DVRGV--------

Chr XP_001701105 DTFLL-ALEEIAAKKRVSVDTLFG----RLADADPLAATTKAEYVRHHDDKSTYTGVYRAGGPRNLAGAR--------------------------------

Vc XP_002946668 AQFCA-ALDHFAQKKGCTQAELAQKVAE----ASPTSNATKAQAVKYYDDKSMFTGVHKNGGPTTVDK--M-RAGGLANLCDRSPA---DNRGVKY------

Chv EFN56830 DAFEQ-ALQKVATKKGVSMAEVVSTIVA---AGGPKSNGTRAEACRFYDDKSNWTSTARNGGPTNVDGQ-----KDLSSLCDRTAA---DARGISAKSNFSR

Mp ACO68100 HDFAL-ALHFVAEEKGTTYETLVAQICERVATHAPDAHGTKAAFTRLHDDKDTYTGAY--------------------------------------------

Pem EER04926 GQFME-ALQEVADRLDKPISWAKEKVVE---CDGPTIRTSVGPSARPSIDAGSDRKVWRNGGPQTVDTGASSQAPA-TEVTKRASG---STSTPAGK-----

Pem EEQ99821 GQFLE-AIGEVWRNGGPRSVDSGRPSQQAIGKTSTAGVTKRATGERFFYDTSTYTGVHKAGGPETIDNHDKGLVEVGSP-----------------------

CLUSTAL 2.1 multiple sequence alignment - short p25alpha domains (Supplementary figure 4)

Os CT850609* LDQVFKNFNAGG----------LEMDNRQFAKVAKDTGILD-KKLTATDVDLIFNKVK

Tae CD868723* LESVFKAFA-GG----------AEMDGRAFVKLTKDTGLLD-KKLTTTDVDLIFAKII

Chr XP_001695016 LKNAFIAFASYGKGQM----MKQDMDNKNFSKCIKDSGILD-KVITSTEVDITFMKVK

Chr XP_001695015 LRLAFIAFASYGKGQE----LKQDMDNKNFSKCIKDSGIMDAKCITATEVDITFMKVK

Chr XP_001701105 AQHVFIKYASIGKTGIT--LANATLDGSKFAKLCRESKLIG-GGLTPMDVEGIFLRVS

Vc XP_002946668 LKEAFIAFASYGKGQE----IKQDMDNKNFSKCMKDSKIIDGKCITNTEVDITFMKVK

Chv EFN56830 LRVVFTAFAAFGSGRDS--GISSGLEGRAFSKIFKDAGLY-CRKFTVTDADLIFTSVK

MpACO68100 ------------------------MESRGFIKMLRDCNVLN-KRFNDAAADIIFTKVK

Pam EW705544* -------------------SHYGRGHLIDKNLTLAIVDLVFNQIKPKGGRTITFKVFC

Pt XP_001436768 VQQVFLQFT----------ANKPEMDGKTFAKVSKDCHLLD-KKLTSTDVDLIFAKIK

Pt XP_001423901 VQQVFLQFT----------ANKPEMDGKTFAKVSKDCHLLD-KKLTSTDVDLIFAKIK

Tth XP_001023601 LEGVFKKFT----------ANKADMDGKTFAKFAKDCGLLD-KKLTATDIDLIFAKVK

Tth XP_001023599 LEGVFKKFT-GG---------KIEMDGKTFAKFAKDTGLLD-KKLTATDVDLIFAKVK

Tth XP_977237 LSAAFKKFT----------NQRSTMDSKTFVNTLADSGIFNFKITTHQSEQIFEKVKN

Tth XP_977235 LAAAFKKFT----------NEKSSMDVKIYLSVLKESGVLNYKVQADLAENFFNTIKN

Tth XP_977236 LLSAFKKFT----------NDRSTMEVKIFIGALTDSGVFNGRVNPSQAEYIFDKVKN

Tth AAL79503 -------------------GDRSTMEVKIFIGALTDSGVFNGRVNPSQAEYIFDKVKN

Bb XP_001610770 LHQIYQHYINKSTG---------QLEGRMFVKIFKQANLLDQKLN-TNDLDIIFVKHR

ETH_00003465 LEELFSNFG-RGK----------DLDSRTFVKLCKDCKLLG-GGLTQTDCDLIFTKCK

ETH_00015030 LEEAFRVYT-KGA---------AEMDGRTFGKMLKDCGVLSSSKMTAVDADLIFAKVK

Nc CBZ50502 LDGVFKSFT----------HDAPAMDGRTFVKLCKDCKVFD-KNYTTTDADLVFTKVK

Nc CBZ53835 ASGAFQVYT----------KGSGDMDGRTFVKILKDTAILDGKTLTTVDADLIFTKVK

Nc CBZ53276 LASVFQSYT----------QGKGDMDSRTLVKLCKETGMID-KQTTATDIDLIFTKCK

Pf XP_001350760 MENAFYVYT----------KNLPDMDSRTFVKILKDAKLLN-KKFTTVDADLIFAKVK

Py XP_726013 METVFNIYT----------KNMPDMDSRTFVKILKDSKLLN-KKITAVDADITFARVK

Pch XP_740774 MEGVFNIYT----------KNMPDMDSRTFVKILKDSKLLS-KKITAVDADLAFAKVK

Pb XP_674367 METVFNIYT----------KNMPDMDSRTFVKILKDSELLN-KKITAVDADITFAKVK

Pv XP_001613902 MENAFYIYT----------KNEADMDSRTFVKILKDAKLLS-KKLTAVDADLTFAKVK

Pk XP_002262452 MENAFYIYT----------KNEADMDSRTFVKILKDSKLLN-KKLTAVDADLTFAKVK

Tha XP_953847 ISKVFDNYK----------DQNNVLKGRMFIKMFKDANLIS-SSSETNNLDLIFTKYK

Thp XP_766449 LSELFERY-----------RDQ-NLKGRMFVKMFRDAGLIT--SYD-NSLDLIFAKYK

Tg XP_002369913 LDAVFKSFT----------HDAPAMDGRTFVKLCKDCKAFD-KNYTTTDADLIFTKVK

Tg XP_002367917 IAGVFQSYT----------QGKGDMDSRTLVKLCKETGVID-KQTTPTDIDLIFTKCK

Pem EER04926 ---MYKAFTGGDN----------MMDGRQFAKLCKDCQIVEKGSLSVNDIDIIFAKVR

Pem EEQ99821 LLEMYKAFTGGDT----------MMDGRQFAKLCRDCNIVDKKGLSVNDTDIVFAKVR

Lm XP_001686248 FQATFEAFASFGSAP------SKEMDNSHFSKMLKECKIIG-KSFTSTDADLLFSKVK

Lm XP_001686249 ------------------------MDNSHFSKMLKECKIIG-KSFTSTDADLLFSKVK

Lb XP_001563405 FQATFEAFASFGSGP------SKEMDNSHFSKMLKECKIIG-KTFTSTDADLLFSKVK

Lb XP_001563404 PETIFEAFASFGSGP------SKEMDNSHFSKMLKECKIIG-KTFTSTDADLLFSKVK

Li XP_001468533 FQATFEAFASFGSAP------SKEMDNSHFSKMLKECKIIG-KSFTSTDADLLFSKVK

Li XP_001468534 ------------------------MDNSHFSKMLKECKIIG-KSFTSTDADLLFSKVK

Tb XP_844424 MEAVFYAFASFGTAP------TKEMDNAHFSKMLKEAKIIG-KTFTSTDADLLFNKIK

Tc XP_806144 IESAFYAFASFGGAP------TKEMDNAHFSKMLKETKVIG-KQFTSTDADLLFNKVK

Al EC630993* ----------------------------------RDTKLLD-KKFTATDADLIFSKAK

Dp EC843061* ------------------------MDNSRFNKFCKDAGIIDGRKFTSTDADLIFTKSK

ChrXP_001691800A LKAIFRDFASFGTR-----QQVEEMDSAHFAKLFRDCGLLG-PDLTLTDIDLAFTAAK

ChrXP_001691800B LYESWLMWQQFGAGAGAGPSRAVEMGPAQYVKLLRETGIITGKDFTSVQAELIYAKVK

VcXP_002948912A LKQFFDDFCSFGTR-----QVVDEMDNVHFAKFCKDCNLLG-RDLTVTDIDLAFARAK

VcXP_002948912B LQDMWLMWANFGTGNNSNPAPRPEMGVAQFIKLVRETGLLD-KHFTAVQAELIFVRAK

OtXP_003078535 MRAAFVQFASFGRGRATISDERGTMDSSRFAKMCRECVFR--DDPESEEKMRAVDVAF

OlXP_001421186A LTRAFDAFGSFGRRR----DAETRMDGARFAKLCKEVIFKGAGDDAREGKLRRAEIAF

OlXP_001421186B VKGLFTAFESFLQFS----PPQSALSCSRWVKICEDCELFEAHGLDHPSAGIVFNAVA

MpXP_003061031A LNRVFLAFANYGSANKK--EKLQHMDGAKWAKFCRDCGLQNSKTFTAVQVDLAFQKVK

MpXP_003061031B LKKLFNAHCAFGK------GDKDLMNQKTFCKVMKDCNLFD-KKFTPTRADIIFTKIK

MpXP_003058058A LFQVYLQHCS--------KKYPQEMGSAQFVRACRKANFID-AMCTASACCIVFAKSR

MpXP_003058058B FKALYVDHCSYGKG----LKKIDEMDGAAFARMFKNANMLDKN-FTSTAVDIIFTK-S

MpXP_003058058C FKELFIAHCSYGKG----QNKIEELDGAAFSRIFKNAGLFDAK-LSATSVDIIFTKV-

MpXP_003063447 LRASFEAFALYGKGSNPDPMYKEMLSSRDYMKMMVDCGLIGGA-VTTTVVDLIFTRL-

MpXP_002506378A LRRSFEAFAGYGSGGNAGDG-ADAVTGRDWAKLVRDCGLIGGS-VNKASCDIIFAR-A

MpXP_002506378B MLAAFEAFG----------GSKHGMRPEAFEATVRACGLIG-AKVTEGTAAVIFAKCK

MpXP_002507907A FLEIFVKHCSFGAG----QKRVEEMASKSFLKCLNEVDVIDNKQLTEAMVDQVFTKFV

MpXP_002507907B FKTLFEEWANFGIPTKK-QKKVAEMQSAGLNKMLMKTGLKK-PGIEHAWMDNVFKKHA

EsCBN76132 LEQIFLDFTSFGAGQ----SGSSEMDSAKFVKLAKDCKLVG-KNLSTTDLDLIFTKVK

EsCBN76131 ------------------------MDSAKFFKLMGDSGLTG-KSLTRTDCDLIFTKTC

EsCBJ49059 MAKEMSRFCVERARLRKIGSGSFRLEGRAFNKLFASTPGVFSSSFLATDVDLVFARVK

AlbugoCCA24272 LKCIFHYYCVWGRRNAKE-SALFMLDHSKFGRIFRDCPHLINAKFPRAAIDLIFFKVL

AlbugoCCA17632 LKGIFHYYCRFGRTAAKG-VDAKTLDNANFSKLCRECPELVDSRLTRTEIDLIFVKVK

PrPhyra80518 LKAVFHYYCRFGRTGAKG-LGEKTLDNSNFVKLCRDCPDLLNASFGKTDVDLIFVKAK

PiXP_002907772 LKAVFHYYCRFGRTGPKG-LGEKTLDNSNFVKLCRDCPDLLNAFFGKTDVDLIFVKAK

PsEGZ26181 LKAVFHYYCRFGRTGPKG-LGEKTLDNSNFVKLCRDCPDLLDSSFGKTDVDLIFVKAK

PiXP_002907084 LWRIFTFYCVNGDS-----MELEYLKAHQFNKLLRDSRVYG-GHLTPAMVDIIYTSET

PiXP_002905233 VDRVLYTVDLAKRQWKRKSGNLVEMDNSHFARFLKPHGFIHKTHFPMQNADVIFAKMK

NgXP_002683090A LEVIFRSISLGNPTISLFYDSILETVSNILQKTLNGEEQIDCLEFRKITAELSQKRSK

NgXP_002683090B LENVFMYYCSFGNR-----MNLDLMSSSKFKMFIRDTNIYN-YGFKQEEADLIFVKIL

NgXP_002682916 LLEIYNYYCTNIIHT---FSDVNEMTSNQFAKFAKDCKITD-KTFHSNDVFLVFEEAT

Os CT850609* ANPAIRKIKYSQFEE-AMTH-FATKKGIKEDALIEMILKK---GGPKFTATKADF------

Tae CD868723* DKTS-KKANFTAFKS-GVVQ-FAAKKGISEADCTALIVKA---GGPKYEGTKADF------

Chr XP_001695016 AKTD-RTINFAQFCT-ALEH-FAAKRGVSVDSLHAKVEA----ASPTSNATQAEA------

Chr XP_001695015 EKTA-RTINFEQFAL-ALES-FASKKGCPVAQLEEKIEG----AQPKNNATIAQAR---TS

Chr XP_001701105 KSFG-R-INYDTFLL-ALEE-IAAKKRVSVDTLFGRLAD----ADPLAATTKAEY------

Vc XP_002946668 AKTD-RTINYAQFCA-ALDH-FAQKKGCTQAELAQKVAE----ASPTSNATKAQA------

Chv EFN56830 PKGG-KRISYDAFEQ-ALQK-VATKKGVSMAEVVSTIVAA---GGPKSNGTRAEA------

MpACO68100 DRGE-RFIDIHDFAL-ALHF-VAEEKGTTYETLVAQICERVATHAPDAHGTKAAF------

Pam EW705544* EG-L-DKLGSYKYPA-EFKQ--GGAAATTPKLVELINKQK----VPVSTGTKAQA------

Pt XP_001436768 PTPAARSITYAQFEK-GLQM-MAEKKGVGVQDVHNQILNA---GGPHFQGTKADA------

Pt XP_001423901 PTPAARSITYAQFEK-GLQM-MAEKKGVGIQDVQNQILNA---GGPHFQGTKADA------

Tth XP_001023601 TSSAVRTITFAQFEK-GLDQ-MATKKGISLDALKEKVTSA---GGPTFTGTKADA------

Tth XP_001023599 GSSAIRCINIRQFEE-GINQ-FAAKKGISAQDLREKVTAS---NGPSYSGTKADA------

Tth XP_977237 SPNL-RGINYQQFET-CLSL-VASQTGCTRQQIEGLVLEY----SKKFTTKQADP------

Tth XP_977235 NPNL-RGISYPQFEQ-CLSQ-IALKNAQTKQLVEGMVLEY----SKKLGAKKSDP------

Tth XP_977236 SANL-RGINYQQFET-CLSQ-IAAQGGMTRQQIEGMLIEQ----SKKLGAKEGDP------

Tth AAL79503 SANL-RGINYQQFET-CLSQ-IAAQGGMTRQQIEGMLIEQ----SKKLGAKEGDP------

Bb XP_001610770 TKGS-RTMDFSGFEKAIQAA--AVALGIDYQEIVERVLKA---GAPVYAG------TETLP

ETH_00003465 AKGA-ARLTFSEFEA-AMEA-VAAKKKCSLEELLQQVGPG---GGPQFRGTAAEA------

ETH_00015030 DKGS-KKISFLQFQE-ALKL-VAAKKGMDLQTLQQKLAAE-GSEGPILTGTKADN------

Nc CBZ50502 AKGA-KTITFAEFEA-AIGL-IAEKKKVSAQELSAQISS---ASGPVYSGTKALP------

Nc CBZ53835 AKGA-KKIDYAQFEE-ALKL-VGEKKKVSTEQIVSKLAS--GETGPILTGTKADN------

Nc CBZ53276 ARGA-KRLTADDFEK-VVEE-IAARKKKPVDEIIQQLCS---SAGPSFSGTKTDA------

Pf XP_001350760 SKGA-KRINYDQFLE-AVKC-IVEKNKLNYDKFVETLCQE-ASKGPILYGTKTEN------

Py XP_726013 TQGS-KRIKYDQFVE-AIKY-ITEKNKLDYDQFVEQLCNE-ASNGPILYGTKAEA------

Pch XP_740774 TKGS-KRIKYDQFVE-AIKH-LTEKYKLDYDQFVGKLCNE-ASNGPILYGTKAEA------

Pb XP_674367 TQGS-KRIKYDQFVE-AIKY-ITEKNKLDYDQFVEKLCNE-ASNGPILYGTKAEA------

Pv XP_001613902 AKGA-KRINYDQFVE-AVKH-LVDKHKLDYDQFVEKLCNE-ASSGPILYGTKAAN------

Pk XP_002262452 AKGA-KRINYDQFVE-AVKH-LVDKHKLDYEKFVETLCNE-ASSGPVLYGTKAAN------

Tha XP_953847 SKFSG--INYDQFLK-SLKD-VSKVLNMEPSELELKLKR---TNGPIYKG------TETQP

Thp XP_766449 SKCSG--INYEQFLK-SLEE-VSRLLDMKVPELKQRLRE---SEGPIYRG------TEPLA

Tg XP_002369913 AKGA-KTITFAEFEA-AIDL-IAEKKKVSAQELAAQISS---ASGPVYSGTKALP------

Tg XP_002367917 AKGA-KRLTCEDFEK-AVEE-IAKRKNKSVDEITQQLCS---SSGPSFSGTKADA------

Pem EER04926 SRGE-RKIEFGQFME-ALQE-VADRLDKPISWAKEKVVE---CDGPTIRTSVGPS-TALPP

Pem EEQ99821 SRGE-RKINFGQFLE-AIGE-VWRNGGPRSVDSGRPSQQAIATGSTSTPAGKVSGPASVPP

Lm XP_001686248 AKEA-RKISFTEFKEKAIPE-IAAKMKKTPADIEAMIAN----AAPKSSGTKADT------

Lm XP_001686249 AKEA-RKISFTEFKEKAIPE-IAAKMKKTPADIEAMIAN----AAPKSSGTKADT------

Lb XP_001563405 AKEA-RRISFTEFKEKAIPE-VAAKMKKTPEEIETMITN----AAPKSNSTKADA------

Lb XP_001563404 AKEA-RRISFTEFKEKAIPE-VAAKMKKTPEEIETMITN----AAPKSNSTKADA------

Li XP_001468533 AKEA-RKISFTEFKEKAIPE-IAAKMKKTPADIEVMIAN----AAPKSSGTKADT------

Li XP_001468534 AKEA-RKISFTEFKEKAIPE-IAAKMKKTPADIEVMIAN----AAPKSSGTKADT------

Tb XP_844424 AKGA-RKITFTEFNTRALPD-IATKLKMTPEQVAEILTK----ASPASNSTKAEA------

Tc XP_806144 AKGA-RKITLSDFVDKAVPE-IASKLKKSAEELIADISS----CSPEARATKADA------

Al EC630993* AKGE-RKINFATFRDKAVPL-IAEKKGCSVDAVVATACGG---GGPSSSGTKADA------

ChrXP_001691800A GKGE-RKLSFDAFLT-ALAT-CAERKGTGLEALVRAIL---GCEGPVARATKADV------

ChrXP_001691800B PQGC-AKITFECFER-ALGL-IAAAKGTSREALEAAITAS---GGPLLTPGKN--------

VcXP_002948912A PKGC-RKLSFEGFLT-ALAE-CAERKGVNLETLVRSVL---ACQGPVARATKAEN------

VcXP_002948912B PKDS-AKLNFEAFER-AMRL-IAEAKGVNLEDVERSVVKS---RGPMLTARQH--------

OtXP_003078535 ARRARRRVDYETFRQLVTED-LAEIVG-DG-ASAMTVAKKLLGATPSVGAAVSPGK-----

OlXP_001421186A ARRAKRKIDYEDFRALVMED-IDRGDDEDGEADAIATCAKLIGCEPSVESALSPGK-----

OlXP_001421186B TANR-KTLDFDAFKTVLHLA--AQRAGSEFLVFARAVAAGEPRVRSALANSPSSP------

MpXP_003061031A TKGE-RRVDFEEFKDAVAMV--AELRGESFADVCELINAK---GGPQSNATRAEY------

MpXP_003061031B AHGE-RVIKLDGFTR-ALQL-VAQEKNASYEDLVEHVC---ACDGPASSGTAALW------

MpXP_003058058A EELQ-KRIKFPGFLRALAQIAEAVANQFERDALLYPIAVHK--DRKLFEGMNDEAPLTTTR

MpXP_003058058B KEKTKRKITYAQFLSSLGLV-CA-HKGIEYTDLVNVLTET---GPPTASG---------AA

MpXP_003058058C KAKSARKIKYDEYLSGLAQA--AATLGITFEELAEKLTAK---GPP---------------

MpXP_003063447 TVAKQLRWDDGSFFEALAAV--AVEHKATFGQVVSRVSQC----RPQSNVKED----FKIT

MpXP_002506378A TGRNSLRWEDGTFLQACAHI--AAEHRVTFGQVASRISQC----APQATNPG------ASA

MpXP_002506378B HLAGNMVMTFEGFAQAMAHV--ASEYSVKFEVVKDRVFKCTAEKARKEAAKKAAAVVRKPW

MpXP_002507907A KDHPRKRLTYNKFLDALAQI-CS-YKDLDFDDIAGYIHDE---GGPKVTGSQGPATPDRSG

MpXP_002507907B EKPNAKVMKYRPFLDCLAEI--AATDKKKLDDFVKAIKEH---------------------

Dp EC843061* GKGE-RRISYSVFASSTLEE-VAKKKAISKDQLVAQITAK--SSAPSSSGTQAEN------

EsCBN76132 DKTA-KKINFDTFVE-AVEL-ISEKLGKSKDDVILLIVSA--KGPTTGAATVADN------

EsCBN76131 SSSGCKKIHYDAFRKFAIAN-LAAKLGADESAVMSKIASV--TGV-SSSGTVAQA------

EsCBJ49059 DKDE-KTLTYAQFAA-ALNV-VAATLFPKTKRFRDFLAFK--AKAARLLELVEGK------

AlbugoCCA24272 KAGE-RRIKYASFLDALRLVAVGKYPKLPLQQSLPKLVATHLARLPCITDLTNDE------

AlbugoCCA17632 KKGE-RRINYARFLDALGMIASEKYPELPLEQSVPKLLGTHLVKLPCIPELTNGK------

PrPhyra80518 KKGE-RRINYARFLDALGMIAIQKYGEVPLESSVPKLLEAHLAHLPCLLEFTDGK------

PiXP_002907772 NKGE-RRINYARFLDALGMIAIQKYGDMPLEA-----------------------------

PsEGZ26181 KKGE-RRINYARFLDALGMIAIQKYGDMPLEASVPKLLEAHLAHLPCLLEFTDGR------

PiXP_002907084 KGKPQGKMNYEEFLNALVKVATQRLRRPEIVSFMSKFLESLVEIFMFYAKSTNASGSYADY

PiXP_002905233 EPKA-KTISFPRFNKAILMLLADTSINKETKGSEPTIQNGKYRKLLESHCTKRLLWRFAIC

NgXP_002683090A KLQDTSHTLLKPYYKTVSNPDLFTSKKEQLQELFSVYSCLNMKSNSTISLAEFLSFTPHKI

NgXP_002683090B SSTKKKSLTFPQFVDCVKEIAKLRKDITPSKAFAQFLLIDVLPKVHRLENQKYLGN----D

NgXP_002682916 KFSPTFKMNFDDFLNGLAFMSFDEDVKVLFRKLLFELVLPNELTDPSVASLWATHQFTFYT

Os CT850609* VKFHDDKNTYTGVHTKGGPTTVDNKIT-------LSNLADRSKAD--VRGVKISK----

Tae CD868723* VKFHDDKSTYTGVYAKGGPTNVDAGRGG--QVSDISQTCDRTSAD--VRGIKK------

Chr XP_001695016 VKFHDDKNLYTGVYKNGGPTNIDK--Q---AAGGLAGHLDRSPAD--VRGVKF------

Chr XP_001695015 AQYHDDKSLYTGVYKNGGPTNVDKGPT---KAGGLASHLDRSPAD--VRGV--------

Chr XP_001701105 VRHHDDKSTYTGVYRAGGPRNLAGAR---------------------------------

Vc XP_002946668 VKYYDDKSMFTGVHKNGGPTTVDK--M---RAGGLANLCDRSPAD--NRGVKY------

Chv EFN56830 CRFYDDKSNWTSTARNGGPTNVD-------GQKDLSSLCDRTAAD--ARGISAKSNFSR

MpACO68100 TRLHDDKDTYTGAY---------------------------------------------

Pam EW705544* NKFHDDKKLYTGVHAKGGPSTNDNR-------ITLSGLANRAPAN--ARGLNK------

Pt XP_001436768 VKFHDDKNLYTGVHANGGPSTIDKNHG------GLNTICDRSQAD--VRGSQKMMKNI-

Pt XP_001423901 VKFHDDKNLYTGVHANGGPSTIDKNHG------GLNTICDRSQAD--VRGVKK------

Tth XP_001023601 VKFHDDKSLYTGVYANGGPSTVDIGNG---KISDISQLCDRTGAD--VRGVKK------

Tth XP_001023599 VRFHDDKSLYTGVYANGGPSTIDIGSG---KISDISQLCDRTSAD--VRGVKH------

Tth XP_977237 SRFFYDKSTYTGVHTKGGPSTLDNNGY-----SQLSQLCDRSSAN--VRGIKN------

Tth XP_977235 NRFFYDKSTYTGVHNNGGPSTIDNNGI-----SHLSQLCDRSGAN--VRGVKF------

Tth XP_977236 SRFFYDKSSYTGVHTKGGPSTIDNNGY-----SHLSQLCDRSGAN--VRGVNK------

Tth AAL79503 SRFFYDKSSYTGVHTKGGPSTIDNNGY-----SHLSQLCDRSGAN--VRGVNK------

Bb XP_001610770 VRFYDDKNSYTGVHAHGGPSVK-------------------------------------

ETH_00003465 VRFYDDKSTFTGVHAHGGPSTVDKKGK---SKITLADICDRSTPD--VRGINKNFK---

ETH_00015030 VRFHDDKSTYTGVHKMGGPTTVDDGRV---QFNDLSKFCDRSEYD--IRGVKKGIIENK

Nc CBZ50502 NKFHDDKSLYTGVHANGGPSTVDGNIN------DISQILDRSAAT--VRGTKM------

Nc CBZ53835 VRFHDDKNTYTGVHKHGGPTLVDEGRT---QFSDLSNICDRSDYD--VRGVKKGVAE--

Nc CBZ53276 VRFYDDKTTFTGVHAHGGPSTVDTPSTKF-AQITLADICDRSTPD--IRGVNKNFQKS-

Pf XP_001350760 VRFFDDKSTFTGVHKQGGPSIIDKNKT---QFSDLSEITDRSEYD--IRGVKMDVAKNV

Py XP_726013 TRFHDDKSTYTGVHKLGGPTTIDKNKT---HFSNISEITDRSECN--IRGVNLSVEKNI

Pch XP_740774 TRFHDDKSTYTGVHKLGGPTTVDKNRT---QFSDISEITDRSECN--IRGVNISVEKNM

Pb XP_674367 TRFHDDKSTYTGVHKLGGPTTIDKNKT---QFSSISEITDRSECN--IRGVNLSVEKNV

Pv XP_001613902 VRFHDDKSTYTGVHKMGGPTTVDKNKT---HFSDISEITDRSECN--IRGVNLSVEKNL

Pk XP_002262452 VRFHDDKSTYTGVHKLGGPTIIDKNKT---HFSDISEITDRSECN--IRGVNINVEKNL

Tha XP_953847 VRLYDDKSLYTGVHLHGGPRIIDK-----------------------------------

Thp XP_766449 VRLHDDKRLYTGVHLHGGPKIGKQ-----------------------------------

Tg XP_002369913 NKFHDDKSLYTGVHANGGPSTVDGNVN------DISQILDRSAAT--VRGTKM------

Tg XP_002367917 VRLHDDKSTFTGVHAHGGPSTVDTPSR---AQISLADICDRSTPD--IRGVNKNFQKS-

Pem EER04926 ARPSIDAGSDRKVWRNGGPQTVDTGAS---VTKRASGSTSTPAGK--------------

Pem EEQ99821 ERFFYDTSTYTGVHKAGGPETIDNHDK---GSP--------------------------

Lm XP_001686248 VRFHDDKSTYTGAAKQGGPTNVDR------NAGSLAGVVDRRQETIDNRGTTAKQI---

Lm XP_001686249 VRFHDDKSTYTGAAKQGGPTNVDR------NAGSLAGVVDRRQETINNRGFTA------

Lb XP_001563405 VRFHDDKSTYTGAAKQGGPTNVDR------NAGSLAGVVDRRQQTIDNRGTTAKQL---

Lb XP_001563404 VRFHDDKSTYTGAAKQGGPTNVDR------NAGSLAGVVDRRQPTLCPRSGKSSTLDSQ

Li XP_001468533 VRFHDDKSTYTGAAKQGGPTNVDR------NAGSLAGVVDRRQPTLCPRSGKSSTLDSQ

Li XP_001468534 VRFHDDKSTYTGAAKQGGPTNVDR------NAGSLAGVVDRRQETIDNRGTTAYKNVKH

Tb XP_844424 VKFHDDKNLYTGVYKAGGPTNVDR------NAGSLSGVVDRRVDQVDVRGTTSSQK---

Tc XP_806144 VKFHDDKNMYTGVYKAGGPTNVDR------NSGSLSGVVDRRVAQTDVRGTTASQK---

Al EC630993* VKFHDDKSLYTGVYARGGPTNVDK------DKITLAGIVSDHDALTDVRGIVRK-----

Dp EC843061* VALHDDKSLYTGVYSKGGPTSNDL------GTSDLSFITNREDAD--VRGVQI------

ChrXP_001691800A VRLHDDKSTYTGVYAKGGPKVTEKAHD-------LAALLDRSDAGAARAGPITIVDKPA

ChrXP_001691800B -----------------------------------------------------------

VcXP_002948912A VRLHDDRSTYTGVYAKGGPKVSDTAHD-------LATLLDRS-FDSNRQSGHLFDSKPQ

VcXP_002948912B -----------------------------------------------------------

OtXP_003078535 CRFHDDKTAYTGVYAERHG---IERSASRRETSPRAAQIDVEKLPTDEAFLQFSPPLAS

OlXP_001421186A VRFHDERERYTGVQAEKHGRGALERAASRRSVETHAAPEEASLPD--------------

OlXP_001421186B LRFHDEVATYTATHKATHRDKVLSP--------RALSIRKRAGIADD------------

MpXP_003061031A VKF-ADPDNFTGAYAANVGRSSVMRHVP---SNWERELKNVKPTPR-------------

MpXP_003061031B TKFHDDKSTYTGAYA-----AIAGASK-------STRVHESHDAWKSKGLRPVFD----

MpXP_003058058A FRPEDDDAIPTLTPAAGPMASTKPKPIPTVTSEALKDLQKRSKPVDMIDDVGQAPVPKS

MpXP_003058058B GPGVSKPEPAKRAKPAKSGPLHPKPSAP---VAALIAKVGE------EGDAASDLPEPK

MpXP_003058058C -----------------------------------------------------------

MpXP_003063447 GAADGKKERVAAASALDAAQQANGPAPPPEEEYQTG--DVLTPVDALLRRMPLEVSQTA

MpXP_002506378A SASAGAEERNAAAAARAAAESANGPVPGPEAASGEAGAQALAPVEALLRCVALDAAQSA

MpXP_002506378B ESKKNNDRAEAEARAAEAAEAAAAAAAA--EAAAKAAADAASADPVQVPGEAYTDAELI

MpXP_002507907A GSGDDRPKAGTQKLPKKAAPAKEKSRINVLDKFDDDGQGQIDRVEFKDKEEGFDFEEEF

MpXP_002507907B GSP---KAKATEVDHLGRPIR--------------------------------------

EsCBN76132 VRFHDDKSTFTGVHNNGGPTNIDGHNV------DMANQLDRSEGAD-VRGVKEYQNDSR

EsCBN76131 SRFHDDKSTYTGSHAAGGPTHVDQR-------VSLESLANRGSAD--VRGVQASVYKLD

EsCBJ49059 VLKAPGATEYRKFCQKAGERFVFRSANK--IQATIRGYLGRKRFRQ-------------

AlbugoCCA24272 ----ELEALFVVE----------------------------------------------

AlbugoCCA17632 ----TVQAVWLRRYSVDN----------------SCVEAPPPPSIEDTSTAAQLPSEDA

PrPhyra80518 ----TVQAVWQKRAEHNE-------------EVVFPPVSPPSPTARLPTAKSSTFDTLD

PiXP_002907772 -----------------------------------------SPTAK--RCVANCS----

PsEGZ26181 ----TVQAVWQKRAEHNEPATADASSPPV-AEEVVAPASPPSPTARLATATATSPGVAM

PiXP_002907084 QRFINDFCFANLQLSSVEAAHVFLASCSSPAQSADCNESGSQTGESVSNGVAAIQRILE

PiXP_002905233 IATKRRHEAFVVIFREQQIQKHRSKCAAT-IQLAYRCYKFRLQLKAMIDHTGRAVKFQH

NgXP_002683090A SRVPLQKTIFYNSLPPEGSESFKESKSKGLSQHPFSNLVKTSKDKVDKDISKFKEFLKK

NgXP_002683090B EKLNVLLDKFRNNLQKAVPPFVGRETE--------QSKVENGGNIVPMTAFSVPTLR--

NgXP_002682916 QKKRTHKDTMQWVSYNKGLSTLNVDEFFMFLAKMFDSCCKINQSFSEGRLMNFVEFLDC

CLUSTAL 2.1 multiple sequence alignment - partial p25alpha domains (Supplementary figure 5)

*Cryptosporidium hominis* XP_666440 NVFDRLLDPKLYTGMHKYRFDKDGNGLGKAGREYLFREDGYTESTKRKHEVV-SSSIK-RHSY-ANISSN

*Cryptosporidium parvum* XP_001388280 SVFDRLLDPKLYTGMHKYRFDKDGNGLGKAGREYLFREDGYTESTKRKHEVV-SSSIK-RHSY-ANISSN

*Toxoplasma gondii* EEA97769 NVFERLTDTAYYTGSHRERFDEFGNGRGIAGREYLYAYDGLTESPSRCHEVY-SSVIK-RPRKPVVTPGT

*Spizellomyces punctatus* SPPG_06588 DVTARLTDPRTYTGTHKQRFDEEGHGRGMAGRKDLVEYDGNTTSAHRGHVPFG-SDQDLRERVDREKPIV

*Trichoplax adhaerens* XP_002111209 DVTSRLTDSSKYTGSHKNRFDDSGKGLGKAGRENMVDYTGSTSSQSRDFAVNK-SNVS-KSDKPVVASAL

*Homo sapiens* NP_008961 TV-SRLTDTTKFTGSHKERFDPSGKGKGKAGRVDLVDESGYVSGYKHAGTYDQ--KVQGGK---------

*Batrachochytrium dendrobatidis* BDEG_06075 SVTQRLTDHTHYTGTHKNRFDEAGQGLGLAGRDTHSRTN-------------ELSKIVNRKE--ADIRGV

*Monosiga brevicollis* Monbr1/23057 GVLDRMTDTSQYTGSHKERFDSEGHGKGLAGRDSTAKGTGHIP-AVGGGS--DLASHLDRSP--ANVRGV

*Jakoba libera* EC692700* EVLEKMTDVNLYSGAHKQRFNEDGTGRGREGRDVIAKGGGTVASAGGSGAVHDLSEIT-RSHLNTGPSAL

*Malawimonas californiana* EC714749* GILSKLTDASQYTGAHKERFDADGKGRGLAGRDH-----------PVTG---DLSELLDRTD--ADVRGV

*Oryza sativa* CT849204* ANVDRMTDTSKYTGAHKERFGDDGKGKGIDGRENRTENSGYVGNYKGANTYDK-AHK-------------

*Chlamydomonas reinhardtii* XP_001690551 SIFDKLTDSSLYTGAHKHRFDASGNGRGLAGRDRVTKGHGFIAGAPG-GSVADLSQIT-RTNLNT-TGAG

*Volvox carteri* XP_002946586 SIFDRLTDSSLYTGTHKHRFDENGNGRGLSGRDRISKGSGFIAGAPGS-AVADLSQIT-RPNLHVASGTG

*Volvox carteri* XP_002946586 SIFDRLNDPSTYTGMHRQRFSSDGRGLGAAGRTMPNAYVSPMDLCR------------------------

Trimastix pyriformis EC840067* SIFDKLTDPKLYTGTHVHRFDNEGHGRGLAGRD-----SGIDSLT------HDLSQMT-RPDAPAHVPHP

Trimastix pyriformis EC840067* SIFDKLTDPKLYTGTHVHRFDAEGHGRGKAGRD-----PGIDSQT------HDLSQMT-RPALDTGATVP

Trimastix pyriformis EC840067* SIFDKLTDSSLYTGTHSQRFDAQGRGRGLAGRD-----SGIDSQT------HDLSQMT-RTDL----GSG

*Thecamonas trahens* AMSG_02233 DITARLTDPSTYSATHKHRFKSDGTGAGKAGRVDVVENDGYVSSYK-----KQKSDGKV-SKRVGGKAAD

*Thecamonas trahens* AMSG_02233 DITARLTDPSTYSATHKHRFRADGRGAGKAGRVDVVENDGYVSSYK-----KQKSDGKV-SKRVGGKAAD

*Thecamonas trahens* AMSG_02233 DITARLTDPSTYSATHKHRFKADGTGAGKAGRVDVVENDGYVGAYR-----EKRRGGKAPSKRVGSKASS

*Thecamonas trahens* AMSG_02233 SVVDRLTDTSGYTGAHKSRFTPDGKGRGKAGRVDVVENTGYTASFD-----KSRSRGRG-SRR-------

*Hyperamoeba dachnaya* HDE00004089* DVTSRLTDPSKYTGAHKNRFDASGKGKGIEGRVDRVSGDGYVTGYTGYTGGKDITPTK-TPSVVDRLTDP

*Hyperamoeba dachnaya* HDE00004089* DIFDRLTDSSKYTGSHKNRFDSSGKGKGIDGRVERSDNNGYVQGSKIAK---------------------

*Monosiga brevicollis* XP_001750206 EVYHRLTNHGSYTGTHKARFGKDGVGLGLEGRRDDDETNVAIAGQAIITREVDD-EM-DRPHRPAMPWEA

Salpigoeca rosetta EGD82798 PLFRRLTDPRLYTGSHKHRFDEQGAGKGLYGRDSIPKGKGIPVSTCLGFETALRTASDVRGVPLATLTAS

*Lolium perenne* GR509039* PIFDKLTDPSQYTGAHRARFDAQGHGLGLAGRTDVPLERFQGDAYAN-ADVRDLAHIT-RPQYNAGSRYA

*Giardia lamblia* XP_001705540 SIFDRLTDPSTYHGTHKERFNADGTGRGLAGRDSVAKGSGTVGG-----RVGDLSSQVSRK---------

*Jakoba libera* EC691986* SIFDRLTDQRTYTGVYAERFKS-GGG-INGHADSG--------------NVTDLSQIT-RPQ--FSGKGS

Jakoba libera EC691986* SIFDRLTDQSTYTGVYAERFKS-GGG-INGHADSG--------------NVTDLSKIT-RPQ--YSGKGS

*Seculamonas ecuadoriensis* EC817264* SVYDRLTDKSKYTGVYAERFKT-GGG-INAHAAGG--------------NVNDLSQIT-RPN----LRGA

*Seculamonas ecuadoriensis* EC817264* SIFDRLTDQSLYTGVYAERFKS-GDGRINSHASNS-------------SGVASLSQIT-RPNLSTSTHMG

*Seculamonas ecuadoriensis* EC817264* NIFDRLTDQSKYTGVYAERFKS-GDGRINMHASGA---------------VCATCRDH-AP---------

*Naegleria gruberi* D2VER9_NAEGR SVFERLNDPSNYTGVYFERFRT-QNGHINANSSSG--------------NVNHLQEIL-RPSVRSSTCVH

*Naegleria gruberi* D2VER9_NAEGR SVFERLNDKQNFTGVYKERFES-GIGSINGSSEYY---------------DNENNHSS-IP---------

*Phytophthora infestans* XP_002907772 NVYSRLYDPKSYTGVYKKRFEA-DCNDLTERV------------------VHDLSNAM-RTNLNHDVNRP

*Phytophthora ramorum* phyra80518 NVYSRLSDPKSYTGVYKKRFEA-DCNDLTERV------------------VHDLSNTM-RTNLNYDVSRS

*Phytophthora sojae* EGZ29591 NVYSRLYDPKSYTGVYKKRFES-DCHDLTERV------------------VHDLSNAM-RTNLNYDVSRP

*Ectocarpus siliculosus* CBN76131 GVYDRLCDQKSYTGVYAERFKGEG-GRINGDT------------------VNDGVAFSGNTNSGGDHAVR

*Chlamydomonas reinhardtii* XP_001695016 -FHD---DKNLYTGVYKN-----G-GPTN---IDKQA-------------AGGLAGHLDRSP--ADVRGV

*Plasmodium falciparum* XP_001350760 -FFD---DKSTFTGVHKQ-----G-GPSI---IDKNKTQ-----------FSDLSEITDRSE--YDIRGV

*Tetrahymena thermophila* XP_001023601 -FHD---DKSLYTGVYAN-----G-GPST---VDIGNGK-----------ISDISQLCDRTG--ADVRGV

*Trypanosoma brucei* XP_844424 -FHD---DKNLYTGVYKA-----G-GP-----TNVDRN------------AGSLSGVVDRRVDQVDVRGT

*Oryza sativa* CT850609* -FHD---DKNTYTGVHTK-----G-GPTT---VDNKIT------------LSNLAD---RSK—ADVRGV
