# Supplementary material for: A New Protein Superfamily: TPPP-Like Proteins
Source: PLoS One. 2012 Nov 14;7(11):e49276. doi: 10.1371/journal.pone.0049276 (PMC3498115; doi:10.1371/journal.pone.0049276)
Supplement: Figure S3 — Multiple sequence alignment of Triticum, Hordea and Oryza TPPPs by ClustalW. The alignment was refined manually. Amino acid residues identical and similar in both long- and short-type TPPPs are indicated by black background. Amino acid residues identical and similar only in long- or short-type TPPPs are indicated by pink and blue backgrounds, respectively. The three pairs of amino acid residues identical and similar only in long- and short-type Oryza TPPPs are indicated by grey background. (DOC) [file pone.0049276.s003.doc]

Triticum short ME-AA------TTLESVFKAF--------------GGAEMDGRAFVKLTKDTGLLD-KKLTTTDVDLIFAKIIDKTS-----------KKANFTAFKSG Oryza short MEPAATTTEVETTLDQVFKNF------NA------GGLEMDNRQFAKVAKDTGILD-KKLTATDVDLIFNKVKANPAI----------RKIKYSQFEEA

Oryza long MASGGG-----SNLHEIFEKYARFGKTEAQIKEAKGGLRIETKNVQKLCKESGVLDAKYPS-QLLDNDIMRVIGKLVTSHPQHYPKGTKTFEREGFETL

Hordeum long MASGG--------LKEVFDKYSRFGKTESQLKEKD--IRIESKNVQKLMKDTGVVDSKYTT-QLLDNDIARVLGKLTSGG--TYAKGIKTFELNGFKQL

Triticum short VVQFA-AKKGISEADCTALIVKAGGPKYEG----TKADFVKFHDDKSTYTGVYAKGGPTNVDAGRGGQVSDISQTCDR--TSADVRGIKK------ 150

Oryza short MTHFA-TKKGIKEDALIEMILKKGGPKFTA----TKADFVKFHDDKNTYTGVHTKGGPTTVDN----KIT-LSNLADR--SKADVRGVKISK---- 156

Oryza long VHQIAESKK-TDYNAIVAKMSSVSGPSLAGTTGVANKANVDRMTDTSKYTGAHKERFGDD-GKGKGIDGRE--NRTENSGYVGNYKGANTYDKAHK 185

Hordeum long VDQIAESKK--SADQIVQQLNSSGGPSLVGVTGTANKDITSRMTDTSQYTGAHKERFDDS-GHGKGKEGRT--DAANNTGYVGNYKGSGTYDXTH- 177
