# Supplementary figures and images for: A New Protein Superfamily: TPPP-Like Proteins
Source: PLoS One. 2012 Nov 14;7(11):e49276. doi: 10.1371/journal.pone.0049276 (PMC3498115; doi:10.1371/journal.pone.0049276)

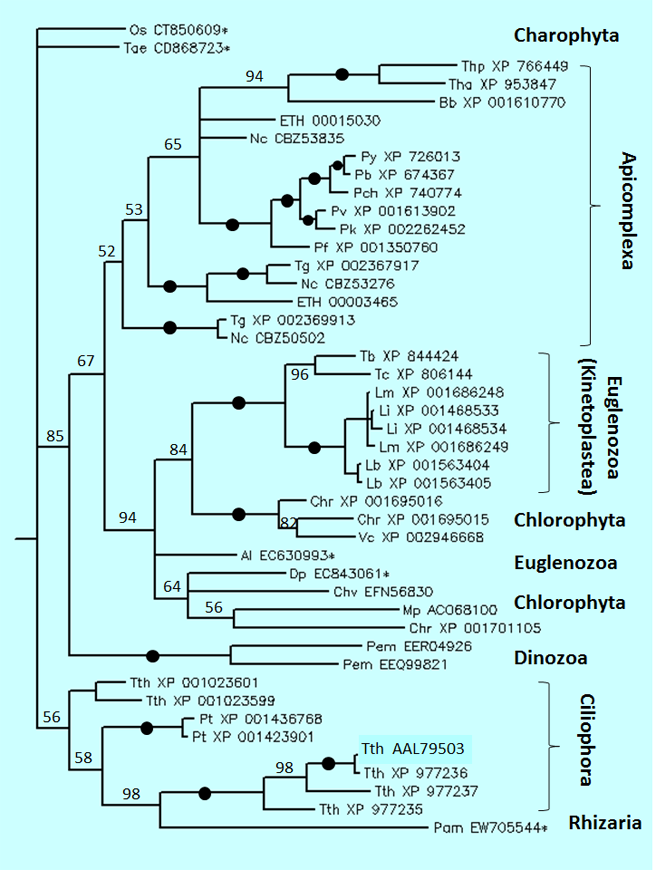

Supplement: Figure S4 — Phylogenetic tree of the short-type TPPPs obtained by Bayesian analysis. Two independent analyses were run with three heated and one cold chain for 2×10^6^ generations, and 1.0×10^6^ generations discarded as burn-in. The numbers at the nodes represent clade credibility values; branches that received maximum support are indicated by full circles. Proteins and ESTs (labeled by asterisk) are indicated by species code and database accession number. ETH (Eimeria tenella) sequences were identified at http://www.genedb.org/. Species codes are: Os, Oryza sativa; Tae, Triticum aestivum; Thp, Theileria parva; Tha, Theileria annulata; Bb, Babesia bovis; Nc, Neospora caninum; Py, Plasmodium yoelii; Pb, Plasmodium berghei; Pch, Plasmodium chabaudi; Pv, Plasmodium vivax; Pk, Plasmodium knowlesi; Pf, Plasmodium falciparum; Tg, Toxoplasma gondii; Tb, Trypanosoma brucei; Tc, Trypanosoma cruzi; Lm, Leishmania major; Li, Leishmania infantum; Lb, Leishmania brasiliensis; Chr, Chlamydomonas reinhardtii; Vc, Volvox carteri; Al, Astasia longa; Dp, Diplonema papillatum; Chv, Chlorella variabilis; Mp, Micromonas pusilla; Pem, Perkinsus marinus; Tth, Tetrahymena thermophila; Pt, Paramecium tetraurelia; Pam, Paracercomonas marina. (TIF) [file pone.0049276.s004.tif]

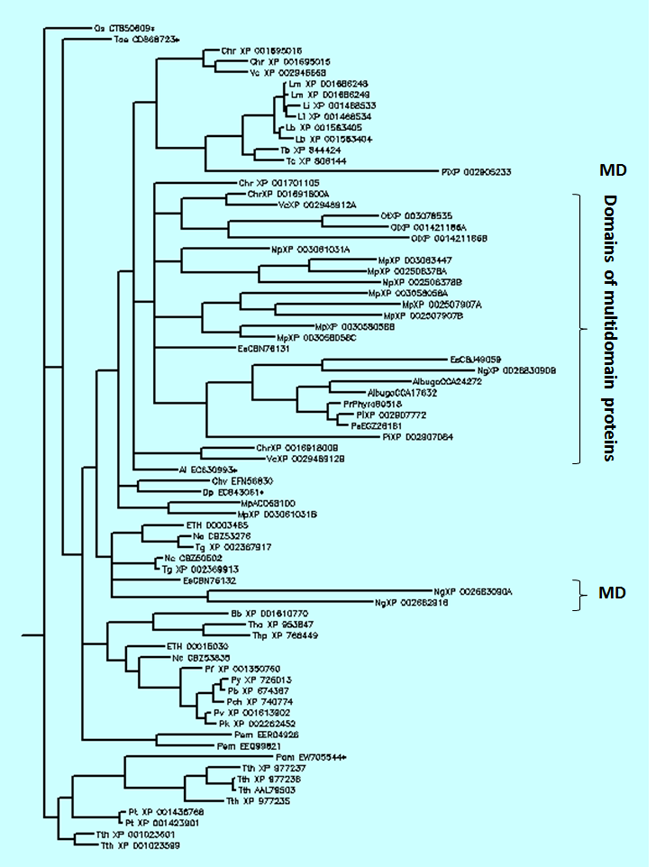

Supplement: Figure S5 — Phylogenetic tree of the short p25alpha domains obtained by Bayesian analysis. Two independent analyses were run with three heated and one cold chain for 2.6×10^6^ generations and 2.1×10^5^ generations discarded as burn-in. Species codes are the same as in Fig. 4 and Figure S4. Further codes are: Ot, Ostreococcus tauri; Ol, Ostreococcus lucimarinus; Es, Ectocarpus siliculosus; Albugo, Albugo laibachii; Pr, Phytophthora ramorum; Pi, Phytophthora infestans; Ps, Phytophthora sojae; Ng, Naegleria gruberi. The Accession Numbers of proteins and ESTs (*) are listed in Figure S1. MD stands for “domains of multidomain proteins”. (TIF) [file pone.0049276.s005.tif]

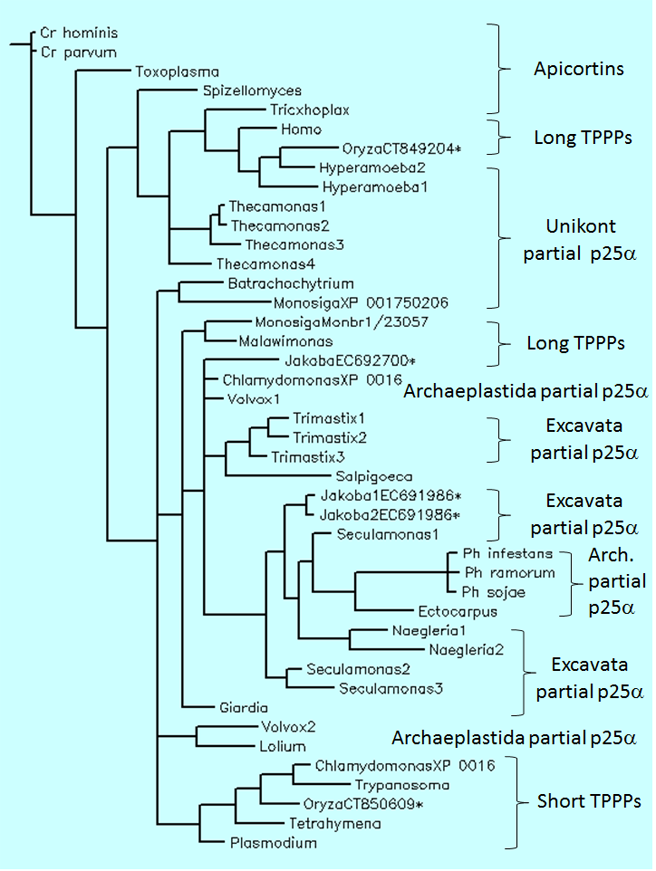

Supplement: Figure S6 — Phylogenetic tree of the partial p25alpha domains obtained by Bayesian analysis. Two independent analyses were run with three heated and one cold chain for 1.1×10^6^ generations and 5.5×10^5^ generations were discarded as burn-in. Cr hominis and Cr parvum stand for Cryptosporidium hominis and Cryptosporidium parvum, respectively; Plasmodium for Plasmodium falciparum, and Tetrahymena for Tetrahymena thermophila. The Accession Numbers of proteins and ESTs (*) are listed in Figure S1. (TIF) [file pone.0049276.s006.tif]

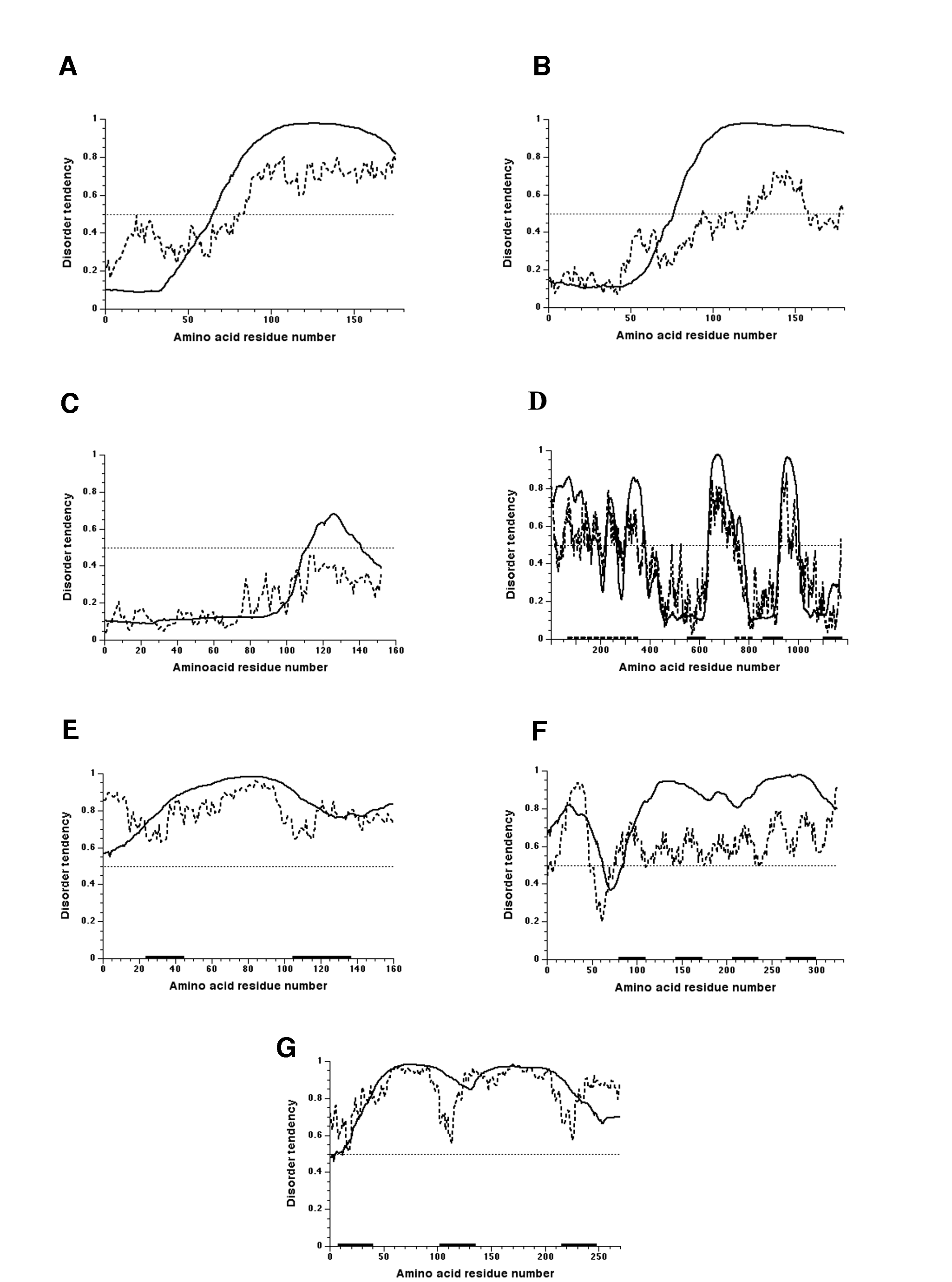

Supplement: Figure S7 — Disorder prediction of TPPP-like proteins using POODLE-L (solid line) and IUPRED (dotted line) predictors. Disorder prediction values for the given residues are plotted against the amino acid residue number. The significance threshold, above which a residue is considered to be disordered, set to 0.5, is shown. A) M. brevicollis (Monbr1/23057); B) S. domuncula (GH560390); C) P. falciparum short-type TPPP (XP_001350760); D) M. pusilla EEH58009 (XP_003058058); E) G. lamblia (XP_001705540); F) T. trahens AMSG_02233; G) T. pyriformis TPE00006173 (EC840067*). The short (D) and partial (E–G) p25alpha and other (COG4942 and EF-hand) (D) domains are indicated by solid and dotted lines, respectively, at the bottom of the plots. (TIF) [file pone.0049276.s007.tif]
