# Supplementary material for: A New Protein Superfamily: TPPP-Like Proteins
Source: PLoS One. 2012 Nov 14;7(11):e49276. doi: 10.1371/journal.pone.0049276 (PMC3498115; doi:10.1371/journal.pone.0049276)
Supplement: Table S1 — Phyletic distribution of the TPPP-like proteins. (DOC) [file pone.0049276.s008.doc]

**Supplementary Table 1. Phyletic distribution of the TPPP-like proteins**

| Domain | Long-type  p25alpha | Short-type p25alpha | | | Partial  p25alpha | | | Partial p25alpha + DCX  DCX | | |  | |
| --- | --- | --- | --- | --- | --- | --- | --- | --- | --- | --- | --- | --- |
| Protein | Long-type TPPP | Short-type TPPP | Multidomain proteins | |  | | | Apicortin | | |  | |
| *Opisthokonta* |  |  |  | |  | | |  | | |  | |
| Choanomonada | Choanoflagellata |  |  | | Choanoflagellata | | |  | | |  | |
| Metazoa | Animals |  |  | |  | | | *Trichoplax* | | |  | |
| Fungi |  |  |  | |  | | |  | | |  | |
| Chytridiomycota | Chytridiomycota |  |  | | Chytridiomycota | | | *Spizellomyces* | | |  | |
| Blastocladio-mycota | *Allomyces* |  |  | |  | | |  | | |  | |
| *Amoebozoa* |  |  |  | |  | | |  | | |  | |
| Mycetozoa |  |  |  | | *Hyperamoeba** | | |  | | |  | |
| *Apusozoa* |  |  | |  | | *Thecamonas*  (*Amastigomonas)* | | |  | | |  |
| *Archaeplastida* |  |  | |  | |  | | |  | | |  |
| Glaucophyta | *Cyanophora** |  | |  | |  | | |  | | |  |
| Chloroplastida |  |  | |  | |  | | |  | | |  |
| Chlorophyta |  | Chlorophyceae  Mamiellophyceae  Trebouxiophyceae | | Chlorophyceae  Mamiellophyceae  Trebouxiophyceae | | | Chlorophyceae | | |  | | |
| Charophyta | *Hordeum**  *Oryza** | *Triticum**  *Oryza** | |  | | | *Lolium** | | | *Nicotiana** | | |
| *Chromalveolata* |  |  | |  | | |  | | |  | | |
| Stramenopiles |  |  | | Oomycetes *Ectocarpus* | | | *Phytophthora*  *Ectocarpus*  *Aureococcus* | | |  | | |
| Alveolata |  | Apicomplexa  Ciliophora  Dinozoa | |  | |  | | | Apicomplexa | | |  |
| *Rhizaria* |  | *Paracercomonas** | |  | |  | | |  | | |  |
| *Excavata* |  |  | |  | |  | | |  | | |  |
| Fornicata |  |  | |  | | *Giardia* | | |  | | |  |
| Jakobida | *Jakoba**  Histionidae*** |  |  | | *Jakoba**  *Seculamonas** | | |  | | |  | |
| Malawimonas | *Malawimonas** |  |  | |  | | |  | | |  | |
| Preaxostyla |  |  |  | | *Trimastix** | | |  | | |  | |
| Heterolobosea |  |  | *Naegleria** | | *Naegleria** | | |  | | |  | |
| Euglenozoa |  | Kinetoplastea  Diplonemea***  Euglenida*** |  | |  | | |  | | |  | |

Asterisks indicate if only EST sequences are available. Truncated TPPPs are present only in Metazoa.
